# Photobiomodulation and photodynamic therapy in the treatment of pressure injuries: a scoping review^*^

**DOI:** 10.1590/1518-8345.7495.4488

**Published:** 2025-02-17

**Authors:** Alexsandra Martins da Silva, Gabriela Machado Silva, Jerusa Celi Martins, Taline Bavaresco, Maria Elena Echevarría-Guanilo

**Affiliations:** 1 Universidade Federal de Santa Catarina, Florianópolis, SC, Brazil.; 2 Scholarship holder at Fundo Estadual de Apoio à Manutenção e ao Desenvolvimento da Educação Superior (UNIEDU/FUMDES), Brazil.; 3 Universidade Federal do Rio Grande do Sul, Porto Alegre, RS, Brazil.; 4 Universidade Federal de Santa Catarina, Departamento de Enfermagem, Florianópolis, SC, Brazil.

**Keywords:** Nursing, Pressure Ulcer, Low-level Light Therapy, Photodynamic Therapy, Nursing Care, Review

## Abstract

**Objective:**

to map the scientific literature on photobiomodulation and photodynamic therapy in the treatment of pressure injuries.

**Method:**

this was a scoping review, as recommended by the Joanna Briggs Institute. It included primary and secondary studies available in full in Portuguese, English and Spanish, published in the last 10 years, from 2014 to 2024, in nine databases.

**Results:**

24 studies were included according to the eligibility criteria. The findings suggest that photobiomodulation and photodynamics can play an important role in tissue repair, size reduction and improvement of clinical indicators in the management of pressure injuries. A diversity of laser types used in photobiomodulation was observed and the most common wavelengths used included 658 nm, 660 nm, 808 nm and 980 nm.

**Conclusion:**

the studies identified show that photobiomodulation and photodynamic therapies have promising results in healing, reducing the size of lesions and improving clinical indicators in the treatment of pressure injuries.

## Highlights

**(1)** Light therapies are effective in treating pressure injuries. **(2)** Optimized healing is a promising result of the therapies studied. **(3)** Reduction in lesion size is a positive effect of the therapies used. **(4)** There is clinical improvement shown in the indicators in the studies reviewed.

## Introduction

According to the National Pressure Injury Advisory Panel (NPIAP), the European Pressure Ulcer Advisory Panel (EPUAP) and the Pan Pacific Pressure Injury Alliance (PPPIA), pressure injury (PI) is characterized as damage to the skin and/or underlying soft tissues, which can occur in any region of the body, especially in areas that coincide with bony prominences^1^. 

The application of intense or prolonged pressure, together with shear forces, can result in the formation of lesions that affect not only the superficial layers of the skin, but can also expose deeper structures such as muscle fascia, tendons and bones. Additional factors, such as nutritional status, microclimate, compromised blood circulation and comorbidities, can contribute to the development and worsening of PI^1-2^. 

Acute hospitalizations, especially in individuals with impaired physical mobility, are considered a risk factor for the development of PI^3^. In addition, PI is a negative indicator of the quality of care provided by the multidisciplinary healthcare team and is considered a potentially preventable adverse event^3-5^.

The presence of a PI triggers a series of biological events aimed at tissue repair in order to remedy the skin damage. However, the physiological mechanism of repair is often inadequate, especially in patients with weaknesses resulting from medical conditions. In this sense, it is essential to adopt additional measures to speed up the healing process and mitigate the risks of associated complications^6^.

Furthermore, the integration of technologies in the health area, such as in the treatment of injuries, not only drives significant advances in the quality of life of patients, but also enriches the repertoire of health professionals, especially nurses, resulting in better quality care^7^. In this context, the use of low-intensity light therapy, known as Low-Level Light Therapy (LLLT), in the form of photobiomodulation (MBF) and/or photodynamic therapy (PDT) in the adjuvant treatment of PI, stands out as an important example of these advances^6^. 

Photobiomodulation therapy is characterized by the use of low-intensity light radiation, the effects of which are triggered by the light itself and not by heat. It consists of infrared, visible, ultraviolet, ionizing radiation, such as X-rays, and gamma radiation. MBF is non-ionizing electromagnetic radiation that differs by wavelength, from red to infrared in the 600-1000 nm range, which reaches different depths of the skin, where it stimulates cellular functions and promotes therapeutic effects^8^. MBF therapy is recognized as a promising approach in the healing process of injuries, acting on various inflammatory and enzymatic mediators, modulating these markers and accelerating the tissue repair process. As a result, there is a reduction in healing times, better inflammatory control and, consequently, a reduction in the experience of pain^9-10^. 

In turn, PDT is the combination of MBF therapy with a photosensitizer, thus promoting photochemical reactions that produce reactive oxygen species, such as singlet oxygen, which applied to an infected lesion causes the destruction of microorganisms, such as bacteria and fungi, through irreversible biological damage to the cell membrane^11-12^. 

Considering the urgent need to base clinical practices on solid scientific data, this study aimed to map the scientific literature on photobiomodulation and photodynamic therapy in the treatment of pressure injuries. This approach seeks not only to add new knowledge to the field, but also to provide relevant support for making informed and effective clinical decisions. 

## Method

### Type of study

This is a scoping review that followed the steps recommended by the Joanna Briggs Institute (JBI)^13^ and the Preferred Reporting Items for Systematic reviews and Meta-Analyses extension for Scoping Reviews (PRISMA-ScR) checklist^14^. The protocol for this scoping review can be found on the international platform Open Science Framework (OSF), DOI: 10.17605/OSF.IO/78NEU.

### Research question

To determine the research question, we used the mnemonic strategy Population, Concept and Context (PCC)^13^ Population (P): refers to people with pressure injuries; Concept (C): photobiomodulation and photodynamic therapy; Context (C): health care levels. Thus, the following question was defined: What is the evidence on photobiomodulation and photodynamic therapy in the healing process of people with pressure injuries at health care levels? 

### Eligibility criteria

This review included primary quantitative, qualitative and mixed-method studies^15^, as well as all types of secondary studies^15^, such as systematic, scoping, integrative and narrative reviews, among others, available in full in Portuguese, English and Spanish. The time period considered covered the last 10 years, from 2014 to 2024, justified on the basis of the need to cover a period recent enough to capture the most up-to-date and relevant studies on the topic in question. This allows for a comprehensive analysis of the available literature and incorporates recent findings that may influence the conclusions of the research.

Articles that did not meet the criteria established for the objective and research question were excluded, as were studies on animal models, *in vitro* and grey literature.

### Search strategy 

The search strategy was developed with the support of a librarian, using the Boolean operators AND and OR in Portuguese, English and Spanish. Various combinations of descriptors obtained from the Health Sciences Descriptors (DeCS) and Medical Subject Headings (MeSH) were explored in order to guarantee the breadth and precision of the search. 

The search took place in January 2024 and the following databases were consulted: Nursing Database (BDENF), Cumulative Index to Nursing and Allied Health Literature (CINAHL), Cochrane Library, Embase, Latin American and Caribbean Health Sciences Literature (LILACS), United States National Library of Medicine/Medical Literature Analysis and Retrieval System Online (PubMed/MEDLINE), Scopus, Scientific Electronic Library Online (SciELO) and Web of Science (Figure 1). 

**Figure 1:** Search strategy. Florianópolis, SC, Brazil, 2024

Database	Search strategy
PubMed/MEDLINE*	(“Low-Level Light Therapy” OR “LLLT^†^” OR “Laser Biostimulation” OR “Laser Phototherapy” OR “Low Level Laser” OR “LowLevel Light” OR “Low Power Laser” OR “Low-Level Laser” OR “Low-Level Light” OR “Low-Power Laser” OR Photobiomodula* OR “Laser Therapy” OR “Laser Therapies” OR Photodynamic*) AND (“Wound Healing”[Mesh] OR “Wound Healing” OR “Wound Healings” OR “Cicatrization” OR “Cicatrix” OR “Cicatrization” OR “Scar” OR “Scarring” OR “Scars” OR epithelializ*) AND (“Pressure Ulcer” OR “Bed Sore” OR “Bed Sores” OR Bedsore* OR “Decubitus Ulcer” OR “Decubitus Ulcers” OR “Pressure Sore” OR “Pressure Sores” OR “Pressure Ulcers”) AND (journal article[Publication Type])
Embase (Elsevier)	(“Low-Level Light Therapy” OR “LLLT^†^” OR “Laser Biostimulation” OR “Laser Phototherapy” OR “Low Level Laser” OR “LowLevel Light” OR “Low Power Laser” OR “Low-Level Laser” OR “Low-Level Light” OR “Low-Power Laser” OR Photobiomodula* OR “Laser Therapy” OR “Laser Therapies” OR Photodynamic*) AND (“Wound Healing” OR “Wound Healings” OR “Cicatrization” OR “Cicatrix” OR “Cicatrization” OR “Scar” OR “Scarring” OR “Scars” OR epithelializ*) AND (“Pressure Ulcer” OR “Bed Sore” OR “Bed Sores” OR Bedsore* OR “Decubitus Ulcer” OR “Decubitus Ulcers” OR “Pressure Sore” OR “Pressure Sores” OR “Pressure Ulcers”)
CINAHL^‡^ (EBSCO)	(“Low-Level Light Therapy” OR “LLLT^†^” OR “Laser Biostimulation” OR “Laser Phototherapy” OR “Low Level Laser” OR “LowLevel Light” OR “Low Power Laser” OR “Low-Level Laser” OR “Low-Level Light” OR “Low-Power Laser” OR Photobiomodula* OR “Laser Therapy” OR “Laser Therapies” OR Photodynamic*) AND (“Wound Healing” OR “Wound Healings” OR “Cicatrization” OR “Cicatrix” OR “Cicatrization” OR “Scar” OR “Scarring” OR “Scars” OR epithelializ*) AND (“Pressure Ulcer” OR “Bed Sore” OR “Bed Sores” OR Bedsore* OR “Decubitus Ulcer” OR “Decubitus Ulcers” OR “Pressure Sore” OR “Pressure Sores” OR “Pressure Ulcers”)
Cochrane Library	(“Low-Level Light Therapy” OR “LLLT^†^” OR “Laser Biostimulation” OR “Laser Phototherapy” OR “Low Level Laser” OR “LowLevel Light” OR “Low Power Laser” OR “Low-Level Laser” OR “Low-Level Light” OR “Low-Power Laser” OR Photobiomodula* OR “Laser Therapy” OR “Laser Therapies” OR Photodynamic*) AND (“Wound Healing” OR “Wound Healings” OR “Cicatrization” OR “Cicatrix” OR “Cicatrization” OR “Scar” OR “Scarring” OR “Scars” OR epithelializ*) AND (“Pressure Ulcer” OR “Bed Sore” OR “Bed Sores” OR Bedsore* OR “Decubitus Ulcer” OR “Decubitus Ulcers” OR “Pressure Sore” OR “Pressure Sores” OR “Pressure Ulcers”)
Scopus (Elsevier)	(“Low-Level Light Therapy” OR “LLLT^†^” OR “Laser Biostimulation” OR “Laser Phototherapy” OR “Low Level Laser” OR “LowLevel Light” OR “Low Power Laser” OR “Low-Level Laser” OR “Low-Level Light” OR “Low-Power Laser” OR Photobiomodula* OR “Laser Therapy” OR “Laser Therapies” OR Photodynamic*) AND (“Wound Healing” OR “Wound Healings” OR “Cicatrization” OR “Cicatrix” OR “Cicatrization” OR “Scar” OR “Scarring” OR “Scars” OR epithelializ*) AND (“Pressure Ulcer” OR “Bed Sore” OR “Bed Sores” OR Bedsore* OR “Decubitus Ulcer” OR “Decubitus Ulcers” OR “Pressure Sore” OR “Pressure Sores” OR “Pressure Ulcers”)
Web of Science (Clarivate Analytics)	(“Low-Level Light Therapy” OR “LLLT^†^” OR “Laser Biostimulation” OR “Laser Phototherapy” OR “Low Level Laser” OR “LowLevel Light” OR “Low Power Laser” OR “Low-Level Laser” OR “Low-Level Light” OR “Low-Power Laser” OR Photobiomodula* OR “Laser Therapy” OR “Laser Therapies” OR Photodynamic*) AND (“Wound Healing” OR “Wound Healings” OR “Cicatrization” OR “Cicatrix” OR “Cicatrization” OR “Scar” OR “Scarring” OR “Scars” OR epithelializ*) AND (“Pressure Ulcer” OR “Bed Sore” OR “Bed Sores” OR Bedsore* OR “Decubitus Ulcer” OR “Decubitus Ulcers” OR “Pressure Sore” OR “Pressure Sores” OR “Pressure Ulcers”)
LILACS^§^	(“Terapia com Luz de Baixa Intensidade” OR “Luz de Baixa Intensidade” OR “Bioestimulação a Laser” OR “Laser de Baixa Intensidade” OR “Laser de Baixa Potência” OR Fotobiomodula* OR “Terapia a Laser” OR “laserterapia” OR Fotodinâmic* OR “Terapia por Luz de Baja Intensidad” OR “Luz de Baja Intensidad” OR “Bioestimulación por Láser” OR “Láser de Baja Potencia” OR “Láser de Bajo Poder” OR “Láser de Baja Intensidad” OR “Láser de Bajo Nivel” OR “Terapia por Láser” OR “Low-Level Light Therapy” OR “LLLT^†^” OR “Laser Biostimulation” OR “Laser Phototherapy” OR “Low Level Laser” OR “LowLevel Light” OR “Low Power Laser” OR “Low-Level Laser” OR “Low-Level Light” OR “Low-Power Laser” OR Photobiomodula* OR “Laser Therapy” OR “Laser Therapies” OR Photodynamic*) AND (“Cicatrização” OR “Cicatriz” OR “Cicatrizes” OR Escara* OR epiteliza* OR “Cicatrización de Heridas” OR “Cicatrización” OR “Cicatrices” OR “Wound Healing” OR “Wound Healings” OR “Cicatrization” OR “Cicatrix” OR “Cicatrization” OR “Scar” OR “Scarring” OR “Scars” OR epithelializ*) AND (“Lesão por pressão” OR “Lesão cutânea” OR “Lesão de pele” OR “Úlcera de Decúbito” OR “Úlcera de Pressão” OR “Úlcera por Pressão” OR “Úlceras por Pressão” OR “Úlcera por Presión” OR “Llaga por Presión” OR “Pressure Ulcer” OR “Bed Sore” OR “Bed Sores” OR Bedsore* OR “Decubitus Ulcer” OR “Decubitus Ulcers” OR “Pressure Sore” OR “Pressure Sores” OR “Pressure Ulcers”)
BDENF^||^	(“Terapia com Luz de Baixa Intensidade” OR “Luz de Baixa Intensidade” OR “Bioestimulação a Laser” OR “Laser de Baixa Intensidade” OR “Laser de Baixa Potência” OR Fotobiomodula* OR “Terapia a Laser” OR “laserterapia” OR Fotodinâmic* OR “Terapia por Luz de Baja Intensidad” OR “Luz de Baja Intensidad” OR “Bioestimulación por Láser” OR “Láser de Baja Potencia” OR “Láser de Bajo Poder” OR “Láser de Baja Intensidad” OR “Láser de Bajo Nivel” OR “Terapia por Láser” OR “Low-Level Light Therapy” OR “LLLT^†^” OR “Laser Biostimulation” OR “Laser Phototherapy” OR “Low Level Laser” OR “LowLevel Light” OR “Low Power Laser” OR “Low-Level Laser” OR “Low-Level Light” OR “Low-Power Laser” OR Photobiomodula* OR “Laser Therapy” OR “Laser Therapies” OR Photodynamic*) AND (“Cicatrização” OR “Cicatriz” OR “Cicatrizes” OR Escara* OR epiteliza* OR “Cicatrización de Heridas” OR “Cicatrización” OR “Cicatrices” OR “Wound Healing” OR “Wound Healings” OR “Cicatrization” OR “Cicatrix” OR “Cicatrization” OR “Scar” OR “Scarring” OR “Scars” OR epithelializ*) AND (“Lesão por pressão” OR “Lesão cutânea” OR “Lesão de pele” OR “Úlcera de Decúbito” OR “Úlcera de Pressão” OR “Úlcera por Pressão” OR “Úlceras por Pressão” OR “Úlcera por Presión” OR “Llaga por Presión” OR “Pressure Ulcer” OR “Bed Sore” OR “Bed Sores” OR Bedsore* OR “Decubitus Ulcer” OR “Decubitus Ulcers” OR “Pressure Sore” OR “Pressure Sores” OR “Pressure Ulcers”)
SciELO^¶^	(“Terapia com Luz de Baixa Intensidade” OR “Luz de Baixa Intensidade” OR “Bioestimulação a Laser” OR “Laser de Baixa Intensidade” OR “Laser de Baixa Potência” OR Fotobiomodula* OR “Terapia a Laser” OR “laserterapia” OR Fotodinâmic* OR “Terapia por Luz de Baja Intensidad” OR “Luz de Baja Intensidad” OR “Bioestimulación por Láser” OR “Láser de Baja Potencia” OR “Láser de Bajo Poder” OR “Láser de Baja Intensidad” OR “Láser de Bajo Nivel” OR “Terapia por Láser” OR “Low-Level Light Therapy” OR “LLLT^†^” OR “Laser Biostimulation” OR “Laser Phototherapy” OR “Low Level Laser” OR “Low Level Light” OR “Low Power Laser” OR “Low-Level Laser” OR “Low-Level Light” OR “Low-Power Laser” OR Photobiomodula* OR “Laser Therapy” OR “Laser Therapies” OR Photodynamic*) AND (“Cicatrização” OR “Cicatriz” OR “Cicatrizes” OR Escara* OR epiteliza* OR “Cicatrización de Heridas” OR “Cicatrización” OR “Cicatrices” OR “Wound Healing” OR “Wound Healings” OR “Cicatrization” OR “Cicatrix” OR “Cicatrization” OR “Scar” OR “Scarring” OR “Scars” OR epithelializ*) AND (“Lesão por pressão” OR “Lesão cutânea” OR “Lesão de pele” OR “Úlcera de Decúbito” OR “Úlcera de Pressão” OR “Úlcera por Pressão” OR “Úlceras por Pressão” OR “Úlcera por Presión” OR “Llaga por Presión” OR “Pressure Ulcer” OR “Bed Sore” OR “Bed Sores” OR Bedsore* OR “Decubitus Ulcer” OR “Decubitus Ulcers” OR “Pressure Sore” OR “Pressure Sores” OR “Pressure Ulcers”)

*PubMed/MEDLINE = United States National Library of Medicine/Medical Literature Analysis and Retrieval System Online; ^†^LLLT = Low-level light therapy; ^‡^CINAHL = Cumulative Index to Nursing and Allied Health Literature; ^§^LILACS = *Literatura Latino-Americana e do Caribe em Ciências da Saúde*; ^||^BDENF = *Base de Dados em Enfermagem*; *¶*SciELO = Scientific Electronic Library Online

### Selection of studies

The identified references were imported into Zotero^®^ in order to store, organize and detect duplicate studies. The Preferred Reporting Items for Systematic Reviews and Meta-Analyses extension for Scoping Reviews (PRISMA-ScR)^14^ was adopted to guide both the inclusion process and the presentation of the selection results, following the four stages of identification, screening, eligibility and inclusion. The selection of studies was carried out after removing duplicates and conducted by two independent reviewers, and any disagreements were resolved through internal discussions. Data extraction from the final sample was carried out using a spreadsheet prepared in Google^®^ Spreadsheets, which enabled clear visualization of the information obtained from the studies selected for the final review sample.

### Data mapping and analysis

A data extraction strategy was defined and adapted according to the JBI manual, in order to select the following relevant information: 1) characterization: author, country, journal, theme, year, title, objectives and type of study; 2) clinical applicability; 3) type of technology used; 4) main results and limitations, information that was organized in the form of tables with narrative content in Microsoft Excel^®^.

### Ethical aspects

As this is a scoping study, it does not need to be assessed by an ethics committee. This research is part of the macro-project entitled: Risk assessment and photobiomodulation therapy for the treatment of PI in people with chronic health conditions, which is funded by the Santa Catarina State Research and Innovation Support Foundation (FAPESC), public call No. 26/2020, financial support term No. 2021TR000432.

## Results

A total of 157 studies were found, 99 of which were excluded due to duplication, four for being animal model studies, two for being *in vitro* studies and one for being grey literature (dissertation). Subsequently, 51 remaining studies were submitted to title and abstract analysis, resulting in the exclusion of 20 because they did not fall within the scope of the effect of MBF and PDT therapy on the healing process of people with pressure injuries, and seven because they did not meet the established objective and research question. 

**Figure 2:**
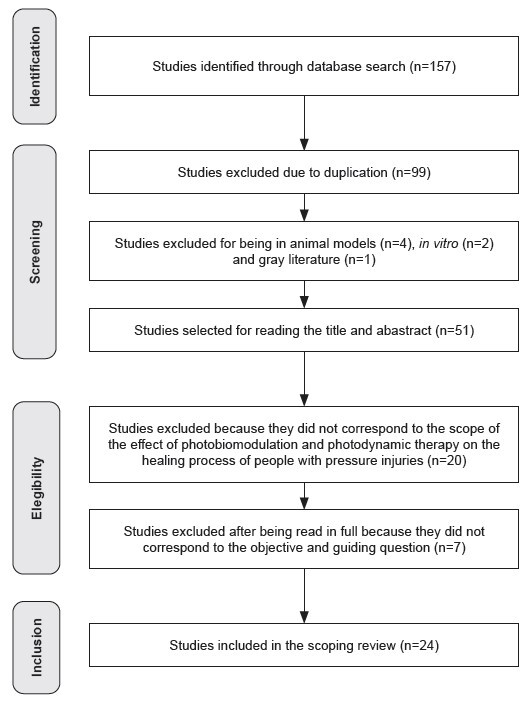
Search flowchart, according to recommendations, adapted from PRISMA-ScR*. Florianópolis, SC, Brazil, 2024

At the end of this process, 24 studies remained that met the inclusion criteria and were selected to be part of this review. These studies were distributed as follows in the databases consulted: PubMed/MEDLINE (n=11), Embase (n=7), CINAHL (n=2), LILACS (n=1), Scopus (n=1), SciELO (n=1) and Web of Science (n=1). No eligible studies were found in the BDENF and Cochrane Library databases after excluding duplicates. The procedure for searching and selecting the studies in this review is shown in the flowchart (Figure 2).

*PRISMA-ScR = Preferred Reporting Items for Systematic Reviews and Meta-Analyses extension for Scoping Reviews

Of the 24 studies included in this review, 8 (33.33%) were case reports, 7 (20.17%) narrative reviews, 5 (20.83%) systematic reviews, 1 (4.17%) experience report, 1 (4.17%) randomized comparative study, 1 (4.17%) randomized placebo-controlled interventional trial and 1 (4.17%) randomized clinical trial. As for origin, 11 (45.83%) were conducted in Brazil, 5 (20.83%) in the United States of America (USA), 2 (8.33%) in Poland, 2 (8.33%) in India, 1 (4.17%) in Italy, 1 (4.17%) in Iran, 1(4.17%) in the United and 1 (4.17%) in Spain (4.17%). In terms of language, 19 (73.33%) studies were in English and 5 (26.67%) in Portuguese. As for the year of publication, 1 (4.17%) was published in 2014, 6 (25%) in 2015, 3 (12.5%) in 2017, 2 (8.33%) in 2018, 2 (8.33%) in 2019, 1 (4.17%) in 2020, 2 (8.33%) in 2021, 4 (16.67%) in 2022 and 3 (12.5%) in 2023. A detailed description of the studies, including title, author, country, year and journal of publication, can be found in Figure 3.

**Figure 3:** Characterization of the studies included in the scoping review. Florianópolis, SC, Brazil, 2024

Id*	Title	Autors	Year	Country	Journal
E^†^1^16^	*Lesão por pressão após COVID-19* ^*‡*^ tratada com laserterapia adjuvante: estudo de caso	Lucena, Pinto, Disconzi, Fabris Mazui, Riquinho.	2023	Brazil	*Revista Gaúcha de Enfermagem*
E^†^2^17^	The role of physical therapies in wound healing and assisted scarring	Fernández-Guarino, Bacci, Pérez González, Bermejo-Martínez, Cecilia-Matilla, Hernández-Bule.	2023	Spain	International Journal Of Molecular Sciences
E^†^3^18^	Photobiomodulation therapy and low-level light therapy in wound healing	Aggarwa, Lio.	2023	USA^§^	Lasers in Medical Science
E^†^4^19^	Effectiveness of electrophysical agents for treating pressure injuries: a systematic review	Vieceli, Martins, Hendler, Santos, Neves, Barbosa, et al*.*	2022	Spain	Lasers in Medical Science
E^†^5^20^	Treatment of chronic wounds with methylene blue photodynamic therapy: a case report	Cesar, Winyk, Santo, Queiroz, Soares, Caetano, et al.	2022	Brazil	Photodiagnosis and Photodynamic Therapy
E^†^6^21^	*Laser de baixa intensidade na cicatrização de lesão por pressão estágio 3: relato de experiência*	Sousa, Soares, Borges, Barreto, Caregnato.	2022	Brazil	*Revista Enfermagem Atual in Derme*
E^†^7^22^	Influence of physiotherapy in the vigilant revitalisation of decubitus ulcer: a case report	Bhagdewani, Sasun, Patil.	2022	India	Journal Of Clinical and Diagnostic Research
E^†^8^23^	Phototherapy (cluster multi-diode 630 nm^||^ and 940 nm^||^) on the healing of pressure injury: a pilot study	Baracho, Chaves, Huebner, Oliveira, Ferreira, Lucas.	2021	Brazil	Journal of Vascular Nursing
E^†^9^24^	Eliminating non-healing wounds: a review	Kuffler.	2021	USA^§^	Regenerative Medicine
E^†^10^25^	Skin surface infrared thermography in pressure ulcer outcome prognosis	Bilska, Stangret, Pyzlak, Wojdasiewicz, Szukiewicz.	2020	Poland	Journal of Wound Care
E^†^11^26^	Effect of photobiomodulation on repairing pressure ulcers in adult and elderly patients: a systematic review	Petz, Félix, Roehrs, Pott, Stocco, Marcos.	2019	Poland	Photochemistry and Photobiology
E^†^12^27^	Photobiomodulation therapy for wound care: a potent, noninvasive, photoceutical approach	Mosca, Ong, Albasha, Bass, Arany.	2019	USA^§^	Advances in Skin & Wound Care
E^†^13^28^	*Efeitos da laserterapia no tratamento de lesões por pressão: uma revisão sistemática*	Bernardes, Jurado.	2018	Poland	*Revista Cuidarte*
E^†^14^29^	Effect of laser therapy on expression of angio-and fibrogenic factors, and cytokine concentrations during the healing process of human pressure ulcers	Taradaj, Shay, Dymarek, Sopel, Walewicz, Beeckman, et al.	2018	Poland	International Journal of Medical Sciences
E^†^15^30^	Application of photodynamic therapy, laser therapy, and a cellulose membrane for calcaneal pressure ulcer treatment in a diabetic patient: a case report	Rosa, Silva, Vieira, Tanajura, Gusmão, Oliveira, et al.	2017	Brazil	Photodiagnosis and Photodynamic Therapy
E^†^16^31^	*Efeitos dos lasers Hélio-Neônio (HeNe* ^*¶*^ ) e Arseneto de Gálio (AsGa**) associados à educação em saúde com foco na promoção da saúde de portadores de úlcera por pressão	Fialho, Baron, Brandenburg, Martins.	2017	Brazil	*Revista Médica de Minas Gerais*
E^†^17^32^	Low-level laser therapy in the treatment of pressure ulcers: systematic review	Machado, Viana, Sbruzzi.	2017	Brazil	Lasers in Medical Science
E^†^18^33^	*Laserterapia em úlcera por pressão: avaliação pelas* **Pressure Ulcer Scale for Healing e Nursing Outcomes Classification**	Palagi, Severo, Menegon, Lucena.	2015	Brazil	*Revista da Escola de Enfermagem da USP*
E^†^19^34^	Improving the ability to eliminate wounds and pressure ulcers	Kuffler.	2015	^USA§^	Wound Repair and Regeneration
E^†^20^35^	Photobiomodulation in promoting wound healing: a review	Kuffler.	2015	^USA§^	Regenerative Medicine
E^†^21^36^	Low-Level laser therapy along with intravascular laser in deep pressure ulcer resistant to conventional therapies	Kazemikhoo, Rahbar, Akrami.	2015	Iran	Journal of Skin and Stem Cell
E^†^22^37^	Nonpharmacologic Interventions to heal pressure ulcers in older patients: an overview of systematic reviews (The SENATOR^††^-ONTOP^‡‡^ series)	Pharm, Lozano-Montoya, Abraha, Cherubini, Soiza, O’Mahony, et al.	2015	Italy	Journal of the American Medical Directors Association
E^†^23^38^	Low-level laser therapy as an antimicrobial and antibiofilm technology and its relevance to wound healing	Percival, Francolini, Donelli.	2015	United Kingdom	Future Microbiology
E^†^24^39^	Closure of chronic non healing ankle ulcer with low level laser therapy in a patient presenting with thalassemia intermedia: Case report	Dixit, Agrawal, Sharma, Singh.	2014	India	Indian Journal of Plastic Surgery

*Id = Identification; ^†^E = Study; ^‡^COVID-19 = Corona Virus Disease 2019; ^§^USA = United States of America; ^||^nm = Nanometer; ^¶^HeNe = Helium-neon; **AsGa = Gallium arsenide; ^††^SENATOR = ENgine Software for the Assessment & Optimization of drug and non-drug Therapy in Older peRsons; ^‡‡^ONTOP = Optimal Evidence-Based Non-drug Therapies in Older People

Figure 4 shows the characterization and synthesis of the articles mapped and included in this review, with specifications on applied MBF and PDT and the main findings of each study.

**Figure 4:** shows the characterization and synthesis of the articles mapped and included in this review, with specifications on applied MBF and PDT and the main findings of each study.

Id*	Objectives	Type of study	Population or articles included (n^†^)	Specifications on FBM^‡^ and/or PDT^§^, and frequency of application	Main findings on FBM^‡^ and PDT^§^ in LP^||^
E^¶^1^16^	To Report on adjuvant laser therapy in a patient with LP^||^ after COVID-19**	Case report	(n^†^=1)	LLLT^††^ with wavelengths of 660 and 808 nm^‡‡^, and 1 J^§§^ red laser and 1 J^§§^ infrared laser.	The results of the healing of the lesions showed healing by second intention and four clinical indicators: evolution of tissue repair with the presence of epithelialization tissue from the 6th and 7th days of application, completely covered granulation, reduced lesion size, and absence of exudate, only transudate.
E^¶^2^17^	To summarize the role of physical therapies as complementary treatments in wound healing.	Narrative review	-		In LP^||^, red light increases healing with better results when compared to LED^||||^ 805 nm^‡‡^.
E^¶^3^18^	To summarize the applications of FBM^‡^ in the field of wound healing and specify the current results in the parameters used for treatment.	Narrative review	-	LLLT^††^ applied seven times.	Results suggest that a specific wavelength of 658 nm^‡‡^, which falls within the range of red light, may be more effective in treating LP^||^.
E^¶^4^19^	To evaluate the efficacy of MBF^‡^, ultrasound and high-frequency electrophysical agents in the healing of pressure injuries in adults and the elderly.	Systematic review	(n^†^=12)		MBF^‡^ showed similar efficacy to other technologies indicated in other studies in the healing of pressure injuries. FBM^‡^ with a red wavelength (660 nm^‡‡^) in stages 2 and 3 of pressure injuries effectively promoted healing compared to standard treatment. It was observed that the use of MBF^‡^ accelerates tissue repair in pressure injuries.
E^¶^5^20^	To investigate the safety and efficacy of PDT^§^ treatment using methylene blue on lesions that showed signs of inflammation indicative of infection.	Case report	(n^†^=3)	-PDT^§^ with methylene blue at a concentration of 10 mg/mL^¶¶^ and LED source^||||^ (660 nm^‡‡^). PDT^§^ applied once a week for three years.	The size reduction observed was extremely significant, going from 26.2 cm*** in length and 9.5 cm*** in width (248.9 cm^2†††^) to 5.2 cm*** by 4.0 cm*** (20.8 cm^2†††^). The infection aspects of the lesion also decreased over the course of the treatment, with the formation of adhered but friable granulation tissue, medium serosanguinolent drainage and a slight odor.
E^¶^6^21^	To report the experiences of a specialist nurse in the management of a clinical case of an elderly patient with LP^||^ stage 3, who underwent low-intensity laser therapy.	Experience report	(n^†^=1)	LLLT^††^ with red (660 nm^‡‡^) and infrared (808 nm^‡‡^) wavelengths with 1 J^§§§^/cm²^††^ red and infrared laser. LLLT^††^ applied every 48 hours for two months.	On the 5th application of the laser, there was a significant improvement in secretion and perilesional erythema. The lesion healed completely after two months of laser therapy and dressings.
E^¶^7^22^	To determine how physiotherapists deal with patients with paraplegia and sacral pressure injuries, as well as the effectiveness of laser therapy in treating large and severe pressure injuries.	Case report	(n^†^=1)	-LLLT^††^ with GaAlAs^‡‡‡^ light, a continuous, non-contact (non-pulsating) beam at a wavelength of 658 nm^‡‡^ of infrared laser. LLLT^††^ applied five times a week, between 8 and 15 minutes of irradiation, for one month.	The treatment of pressure lesions with laser therapy at a wavelength of 658 nm^‡‡^ appeared to be successful.
E^¶^8^23^	To evaluate the efficacy of a prototype LED phototherapy device^||||^ in participants with pressure injuries.	Randomized, placebo-controlled interventional trial	(n^†^=15)	LLLT^††^ with LED^||||^ with a wavelength of 630 nm^‡‡^ (red) and 940 nm^‡‡^ (infrared), a dose of 6 J^§§§^/cm^2†††^ (group I) and 8 J^§§§^/cm^2†††^ (group II). LLLT^††^ was applied three times a week for eight weeks, totaling 24 sessions for each participant.	The area of the LP^||^ showed a statistically significant reduction (p<0.001) over the 24 sessions in all treatment groups. The groups receiving LED phototherapy^||||^ (I and II) showed greater healing compared to group III (placebo). The healing rate was higher in group II, ranging from 94.5% to 98.7% at the 24th session.
E^¶^9^24^	To analyse the etiology of non-healing injuries and different treatments for injury management.	Narrative review	-		Wavelengths of 633-904 nm^‡‡^ cause the fastest healing of LP^||^. LLLT^††^ irradiation also induces healing due to its antibacterial effect. Although 650 nm^‡‡^ is more effective, the bacterial load decreases under treatment with wavelengths of 830 and 904 nm^‡‡^, but increases under the wavelength of 670 nm^‡‡^.
E^¶^10^25^	To evaluate the usefulness of SSIT^§§§^ as a prognostic tool in the treatment of LP^||^ stages III and IV, with hydrocolloid/hydrogel dressings plus 20 exposures to LLLT^††^, compared to hydrocolloid dressings alone, in a group of long-term bedridden patients.	Randomized comparative study	(n^†^=43)	LLLT^††^ with a wavelength of 808 nm^‡‡^. Group I: LP^||^ treated with specialized dressings and laser therapy (five times a week for four weeks); Group II: LP^||^ treated with specialized dressings without laser therapy.	In the study, three variants of LP^||^ healing were observed: pure healing with minimal granulation; healing with hypergranulation; and non-healing. Analysis of the thermographic patterns related to SSIT^§§§^ revealed their dependence on the course of healing. The percentage of successful healing of LP^||^ reached 79.2% in group I compared to 73.7% in group II (p<0.05). The dominant healing variant in Group I was pure healing with minimal granulation, while in Group II the variants pure healing with minimal granulation and healing with hypergranulation were present with equal frequency.
E^¶^11^26^	To conduct a systematic review evaluating the efficacy of MBF^‡^ in the form of LLLT^††^ in the treatment of pressure injuries in adults and the elderly.	Systematic review	(n^†^=5)		FBM^‡^ at infrared wavelength showed efficacy in pressure injury healing, similar to the standard care presented in the different studies. FBM^‡^ (658 nm^‡‡^) was effective in promoting healing when compared to standard treatment.
E^¶^12^27^	To provide background and examine evidence for the therapeutic application of light energy treatments for wound healing.	Narrative review	-		The 658 nm^‡‡^ laser treatment was more effective (70% closure, p< 0.05) in promoting LP^||^ closure. In contrast, the 808 and 940 nm^‡‡^ laser treatments (31% and 30% closure, respectively) did not appear to significantly improve healing rates compared to the placebo group (28% closure).
E^¶^13^28^	To study the effectiveness of laser therapy in the healing process of pressure injuries.	Systematic review	(n^†^=11)		It should be noted that doses of 4 J^§§/^cm^2†††^ with a wavelength of 658 nm^‡‡^ were the most effective in treating pressure injuries.
E^¶^14^29^	To evaluate the effect of laser irradiation at different wavelengths on the expression of growth factors and selected inflammatory mediators in specific phases of the wound healing process. The study included patients diagnosed with chronic lesions of LP^||-^related etiology (II, III, IV).	Randomized clinical trial	(n^†^=67)	LLLT^††^ with GaAlAs^‡‡‡^ light. Group A - 940 nm^‡‡^; Group C - 658 nm‡^‡;^ Group B - 808 nm^‡‡^; Group D - simulated therapy. Treated with LLLT^††^ and analyzed once a day, five days a week for one month.	For group C (658 nm^‡‡^), the change in TNF-α^||||||^ concentration was more intense (reduction of approximately 75%), while the changes in other groups were not so obvious (reduction of approximately 50%). It seems that the successful effect of wound healing after irradiation at wavelengths of 658 nm^‡‡^ is associated with an anti-inflammatory effect, as well as the stimulation of phenomena such as angiogenesis, proliferation or tissue remodeling during the wound closure process.
E^¶^15^30^	To present a case report of LP^||^ in the calcaneus region in a diabetic patient treated with a combination of FBM^‡^, laser therapy and the application of a cellulose membrane.	Case report	(n^†^=1)	LLLT^††^ with a 660 nm^‡‡^ laser (visible red) in a punctual and continuous manner; and PDT^§^ (plate of 30 LEDs^||||^ with a wavelength of 450±10 nm^‡‡^ visible blue for 12 minutes with an irradiance of 30 mW^¶¶¶^/cm^2†††^) and a 1.5% curcumin photosensitive agent.	Healing of the LP^||^ occurred after 30 days of treatment after observation of total epithelialization of the lesion.
E^¶^16^31^	To compare the action of HeNe**** and AsGa^††††^ lasers on the healing process of LP^||^ and to develop a study with preventive measures as treatment.	Case report	(n^†^=3)	Case I - LLLT^††^ with HeNe**** laser; Cases II and III - LLLT^††^ with AsG^a^ laser^††††^. Case I - a total of 52 sessions of LLLT^††^ with HeNe****; Cases II and III - 22 to 46 sessions of LLLT^††^ with AsGa^††††^.	Patient 1 - at the end of the applications, there was a 100% reduction in the lesion and the user reported a substantial improvement in their quality of life. Patient 2 - complete healing of the lesion in 22 sessions. Patient 3 - there was a reduction in the depth of the lesion to 0.5 cm***, with the height and width remaining the same. There was little improvement in the healing of the pressure sore, but a small improvement in the appearance of the sore.
E^¶^17^32^	To evaluate the effects of LLLT^††^ on LP^||^ in humans by means of a systematic review of randomized studies.	Systematic review	(n^†^=4)	-	Significant results were observed when using LLLT^††^ with a wavelength of 658 nm^‡‡^, and no evidence was found for using wavelengths above that for the treatment of LP^||^.
E^¶^18^33^	To describe the healing process of pressure injuries in critically ill patients treated with conventional dressing therapy plus low-intensity laser therapy, as assessed by PUSH^‡‡‡‡^ and by the NOC^§§§§^ injury healing outcome: second intention.	Case report	(n^†^=1)	LLLT^††^ with the AlGaInP^||||||||^ laser, wavelength 660 nm^‡‡^. LLLT^††^ applied once a day, three times a week, for a period of five consecutive weeks, totaling 15 applications.	The lesion shrank from 7 cm*** in length to 1.5 cm*** and from 6 cm*** in width to 1.1 cm***, when comparing the first and 15th day of assessment. The epithelial tissue remained in ascendancy, with a significant reduction in the amount of serosanguinous secretion and the absence of a foul odor. However, erythema and maceration perilesional worsening, probably due to diaper diuresis, which increased perineal moisture.
E^¶^19^34^	To summarize and critically evaluate the evidence from Systematic Reviews on non-pharmacological interventions to treat pressure injuries in elderly patients.	Narrative review	n^†^=110	-	LLLT^††^ and LED illumination^||||^ are used on various types of lesions including healing of non-healing lesions such as diabetic foot lesions, pressure lesions, venous lesions and post-chemotherapy against radiation lesions. In comparative studies, exposure of chronic non-healing pressure injuries that do not respond to standard medical care to wavelengths in the range of 635 and 810 nm^‡‡^ results in their faster healing.
E^¶^20^35^	To analyse research related to the induction of pressure injury healing, gene activation and pain elimination by the application of photobiomodulation and its mechanisms of action.	Narrative review	-	-	People with LP^||^ grade II undergoing 12 weeks of monochromatic pulse FBM^‡^ show a reduction in lesion size of 80 *versus* 50% for patients in the control group, and irradiation with 660 and 880 nm^‡‡^ reduces the initial lesion area by 13 times *versus* control subjects.
E^¶^21^36^	To describe the treatment of chronic pressure injury in patients with spinal cord injury using LLLT^††^	Case report	(n^†^=1)	LLLT^††^ with a wavelength of 980 nm^‡‡^ continuous 6 J^§§^/cm^2††^ for the margins and 655 nm^‡‡^, continuous 1.8 J^§§^/cm^2††^ for the lesion bed together with intravascular laser therapy. LLLT^††^ was applied every other day for 12 sessions and then twice a week for a total of 24 sessions.	After two sessions of laser therapy, the perfusion of the lesion improved and after the 12th session the diameter of the lesion had reduced to 3×5 cm^2††^ with a depth of 1 cm***. After 24 sessions of LLLT^††^ and zetaplasty surgery, the lesion healed completely.
E^¶^22^37^	To summarize and critically evaluate the evidence from systematic reviews of primary studies on non-pharmacological interventions to treat LP^||^ in elderly patients.	Systematic review	(n^†^=45)	-	The level of evidence is very low or insufficient to support the use of adjuvant therapy (ultrasound, negative pressure, laser, electromagnetic, light, shockwave, hydrotherapy, radiofrequency or vibration therapy) to increase the healing rates of LP^||^ in elderly patients.
E^¶^23^38^	To identify the literature that reports only LLLT^††^, without photodynamic agents, as an antimicrobial/antibiofilm technology and to determine its effects on lesion healing.	Narrative review	-	-	Patients with LP^||^ treated with infrared light (956 nm^‡‡^) and red light (637 nm^‡‡^) had a 49% higher healing rate compared to controls. The time taken for the lesion to close by 50% and 90% was significantly reduced. After 5 weeks of treatment, the average lesion area decreased to 10%, while controls took 9 weeks to achieve this result. Few clinical trials have defined a standard protocol for eradicating LLLT^††^ infection. From the available pilot studies, exposing the patient to light of 870 nm^‡‡^/930 nm^‡‡^ and energy doses of more than 100 J^§§/^cm^2†††^ seems to be an effective therapeutic approach.
E^¶^24^39^	To verify the effect of LLLT^††^ in the form of LED^||||^ on a chronic non-healing lesion lasting 6 months in an 18-year-old male patient suffering from thalassemia.	Case report	(n^†^=1)	LLLT^††^ in the form of LED^||||^. LLLT^††^ with a dosage of 17.3 J^§§^/cm^2†††^ for 8 minutes for 2 weeks followed by a proliferative dosage of 8.65-4.33 J^§§^/cm^2††^ for 4 minutes from week 3 to week 6.	On the 6th week of application, the lesion closed.

*Id = Identification; ^†^n = Number of participants; ^‡^FBM = Photobiomodulation; ^§^PDT = Photodynamic therapy; ^||^LP = Pressure injury; ^¶^E = Study; **COVID-19 = Corona Virus Disease 2019; ^††^LLLT = Low-level light therapy; ^‡‡^nm = Nanometer; ^§§^J = Joule; ^||||^LED = Light-emitting diode; ^¶¶^mg/mL = Milligrams per milliliter; ***cm = Centimeters; ^†††^cm^2^ = Square centimeters; ^‡‡‡^GaAlAs = Gallium-aluminum arsenide; ^§§§^SSIT = Infrared thermography of the skin surface; ^||||||^TNF-α = Tumor necrosis factor alpha; ^¶¶¶^mW = Milliwatt; ****HeNe = Helium-neon; ^††††^AsGa = Gallium arsenide; ^‡‡‡‡^PUSH = Pressure Ulcer Scale for Healing; ^§§§§^NOC = Nursing Outcomes Classification; ^||||||||^AlGaInP = Aluminum-gallium-indium-phosphorus

## Discussion

The 808 nm laser therapy resulted in a dominant pure healing variant (H), while the 658 nm laser showed a more significant reduction in the concentration of TNF-α, suggesting an anti-inflammatory effect and stimulation of the healing process^25,29^. 

There is a diversity of types of lasers used in LLLT, as shown in Figure 4. In a case report^22^ where the laser was applied five times a week, between 8 and 15 minutes of irradiation, for one month, and in a randomized clinical trial^29^ with 67 patients who were treated and analyzed once a day, five days a week for one month, the Gallium Aluminum Arsenide (GaAlAs) laser was used, a type of semiconductor laser that was introduced to the market around 1987^40^. Another case report^33)^ used the Aluminum-Gallium-Indium-Phosphorus (AlGaInP) laser, which was applied three times a week for a total of 15 applications. Additionally, in the case report study^31^ which followed three patients, the Helium-Neon (HeNe) laser was used, belonging to the first generation of lasers developed between 1975 and 1985, and the Gallium Arsenide (AsGa) laser, which was the first diode laser available on the market, around 1985^40^. 

In addition, the randomized, placebo-controlled interventional trial^23)^ with 15 participants, who received laser applications three times a week for eight weeks, totaling 24 sessions, showed a statistically significant reduction in the area of the LPs over the course of the light-emitting diode (LED) phototherapy sessions, with the group treated with red LED showing the highest healing rate. These findings suggest that different laser and LED wavelengths can influence LP healing in different ways, emphasizing the importance of selecting the most appropriate treatment for each clinical case. 

The period between 1995 and 2005, known as the third generation, witnessed the introduction of the LED, a semiconductor device with wavelengths ranging from 180 nm to 1 mm. These devices, mainly produced by the spontaneous emission process, were designed to be more affordable and easy to operate electronically^40^. This type of device was also used in three case reports^20,30,39^, which showed good results, with one lesion healing in 6 weeks^39^, another after 30 days^30^ and the third showing a significant reduction in the size of the lesion and an improvement in the appearance of the lesion^20^. In the latter two, PDT was used with photosensitizers such as methylene blue at a concentration of 1%^20^ and curcumin at 1.5%^30^, resulting in a reduction in the size of the lesion, resulting in a reduction in the size of the lesion and the formation of granulation tissue.

The case studies reviewed present a series of encouraging results in the use of laser therapy in the treatment of PI. Firstly, the efficacy of different laser therapy protocols in reducing lesions and improving patients’ quality of life stands out^31^. In addition, laser therapy has been shown to be effective in reducing the size of lesions, improving epithelial tissue and reducing the infection aspects of the lesion^20,22,33^.

Another common point among the studies was the observation of complete healing of LPs after a variable number of laser therapy sessions, indicating the significant potential of this therapeutic approach^21,36,39^. 

The results also indicated that laser therapy can promote healing by second intention, with a reduction in the size of the lesion and improvement in clinical indicators, such as the presence of epithelialized tissue and absence of exudate^16,30^. These findings suggest that laser therapy can play an important role in tissue repair and the management of pressure injuries.

In addition to the findings mentioned above, it is important to note that the studies reviewed used a variety of wavelengths in LLLT. Common wavelengths include 658 nm, 660 nm, 808 nm and 980 nm, each with specific properties that can influence therapeutic results.

Narrative and systematic reviews highlight the efficacy of different wavelengths in LLLT for the treatment of PI. The 658 nm wavelength emerges as one of the most effective, demonstrating a significantly higher closure rate compared to other wavelengths, such as 808 nm and 940 nm^27^. In addition, red light, especially in the 633-904 nm wavelength range, has shown promising results in accelerating LP healing^17,24^. Infrared light therapy, specifically at 637 nm, also showed a significantly higher healing rate compared to the control groups^38^.

On the other hand, systematic reviews highlight the effectiveness of the 658 nm laser at specific doses, such as 4 J/cm^2^, for the treatment of PI^28,32^. In addition, red wavelength MBF (660 nm) in stages 2 and 3 of PI^19^ effectively promoted healing compared to standard treatment^19,26^. However, evidence on adjuvant therapies, such as laser therapy, to increase healing rates of PI in elderly patients is limited and of very low quality^37^.

It is also noteworthy that, according to the reviews, wavelengths of 650 nm, 660 nm and 880 nm showed promising results in reducing the initial size of the LP and promoting healing^18,34-35^. These findings reinforce the importance of considering a variety of wavelengths in laser therapy for PI, highlighting the need for further research to develop standardized therapeutic protocols and treatment guidelines.

It is important to note that this study sought to map the available evidence on BMF and PDT in the treatment of PI, showing that they have promising results in healing, reducing the size of lesions and improving clinical indicators in the treatment of PI. Thus, the results of this study can contribute to the formulation of clinical protocols, guiding investment in technological innovation in health, and consequently contributing to the quality of life of people with PI. 

It is imperative to highlight as a limitation of the research the scarcity of robust in vivo clinical studies on the phenomenon investigated, which results in significant gaps in relation to the most reliable parameters for the use of MBF and PDT therapies in the treatment of PI. It is therefore essential to invest more in research that can establish clear guidelines and standardized protocols in order to provide more solid guidance for clinical practice and maximize the therapeutic benefits of these therapies.

## Conclusion

In summary, the studies reviewed provide a comprehensive overview of the effect of MBF and PDT in the treatment of PI, highlighting promising results in terms of healing, reduction in lesion size and improvement in clinical indicators. The diversity of therapeutic approaches available, including different wavelengths and treatment protocols, highlights the importance of a personalized approach to optimize clinical results.

It is important to note that, despite promising advances, there are still challenges to be faced in the clinical application of these therapies. The lack of consensus on the ideal treatment parameters, such as the choice of the most suitable wavelength and the ideal energy dosage, represents a significant barrier. In addition, the heterogeneity of the studies and the lack of standardization in the methods for evaluating the results make it difficult to compare the different clinical trials. These issues highlight the pressing need for further research, as well as interdisciplinary collaboration between healthcare professionals, to improve the efficacy and clinical applicability of MBF and PDT in LP. 
