# Supplementary material for: Photobiomodulation and photodynamic therapy in the treatment of pressure injuries: a scoping review
Source: Rev Lat Am Enfermagem. 2025 Feb 17;33:e4488. doi: 10.1590/1518-8345.7495.4488 (PMC11835006; doi:10.1590/1518-8345.7495.4488)
Supplement: Translation

# Translation: La terapia de fotobiomodulación y fotodinámica en el tratamiento de lesiones por presión: revisión de alcance^*^

**DOI:** 10.1590/1518-8345.7495.4487

**Authors:** Alexsandra Martins da Silva, Gabriela Machado Silva, Jerusa Celi Martins, Taline Bavaresco, Maria Elena Echevarría-Guanilo

**Affiliations:** 1 Universidade Federal de Santa Catarina, Florianópolis, SC, Brasil.; 2 Becaria del Fundo Estadual de Apoio à Manutenção e ao Desenvolvimento da Educação Superior (UNIEDU/FUMDES), Brasil.; 3 Universidade Federal do Rio Grande do Sul, Porto Alegre, RS, Brasil.; 4 Universidade Federal de Santa Catarina, Departamento de Enfermagem, Florianópolis, SC, Brasil.

**Keywords:** Enfermería, Úlcera por Presión, Terapia por Luz de Baja Intensidad, Terapia Fotodinâmica, Atención de Enfermería, Revisión

## Abstract

**Objetivo:**

mapear la literatura científica sobre la terapia de fotobiomodulación y fotodinámica en el tratamiento de lesiones por presión.

**Método:**

se trata de una revisión de alcance, conforme lo recomendado por el *Joanna Briggs Institute*. Se incluyeron estudios primarios y secundarios disponibles en su totalidad en los idiomas portugués, inglés y español, publicados en los últimos 10 años, de 2014 a 2024, en nueve bases de datos.

**Resultados:**

se incluyeron 24 estudios siguiendo los criterios de elegibilidad. Los hallazgos sugieren que la fotobiomodulación y la fotodinámica pueden desempeñar un papel importante en la reparación tisular, reducción del tamaño y mejora de los indicadores clínicos en el manejo de lesiones por presión. Se observó una diversidad de tipos de láseres utilizados en la fotobiomodulación, y las longitudes de onda más comúnmente empleadas incluyeron 658 nm, 660 nm, 808 nm y 980 nm.

**Conclusión:**

los estudios identificados muestran que las terapias de fotobiomodulación y fotodinámica presentan resultados prometedores en la cicatrización, reducción del tamaño de las lesiones y mejora de los indicadores clínicos en el tratamiento de lesiones por presión.

## Destacados

**(1)** Las terapias de luz son eficaces en el tratamiento de lesiones por presión. **(2)** La cicatrización optimizada es un resultado prometedor de las terapias estudiadas. **(3)** La reducción del tamaño de las lesiones es un efecto positivo de las terapias utilizadas. **(4)** Se observa una mejora clínica en los indicadores presentados en los estudios revisados.

## Introducción

Según el *National Pressure Injury Advisory Panel* (NPIAP), el *European Pressure Ulcer Advisory Panel* (EPUAP) y la *Pan Pacific Pressure Injury Alliance* (PPPIA), la lesión por presión (LP) se caracteriza como un daño en la piel y/o en los tejidos blandos subyacentes, que puede ocurrir en cualquier región del cuerpo, especialmente en áreas que coinciden con prominencias óseas^1^. 

La aplicación de presión intensa o prolongada, junto con fuerzas de cizallamiento, puede resultar en la formación de lesiones que afectan no solo las capas superficiales de la piel, sino que también pueden exponer estructuras más profundas como la fascia muscular, tendones y huesos. Factores adicionales, como el estado nutricional, el microclima, la circulación sanguínea comprometida y las comorbilidades, pueden contribuir al desarrollo y agravamiento de las LP^1-2^. 

Las hospitalizaciones agudas, especialmente en individuos con movilidad física reducida, se consideran un factor de riesgo para el desarrollo de LP^3^. Además, las LP representan un indicador negativo de la calidad de la asistencia brindada por el equipo de salud multidisciplinario, considerándose eventos adversos potencialmente evitables^3-5^. 

La presencia de una LP desencadena una serie de eventos biológicos destinados a la reparación tisular, con el fin de remediar el daño cutáneo. Sin embargo, el mecanismo fisiológico de reparación a menudo resulta inadecuado, especialmente en pacientes con debilidades derivadas de condiciones médicas. En este sentido, se vuelve imprescindible la adopción de medidas adicionales para acelerar el proceso de cicatrización y mitigar los riesgos de complicaciones asociadas^6^. 

Asimismo, la integración de tecnologías en el área de la salud, como en el tratamiento de lesiones, no solo impulsa avances significativos en la calidad de vida de los pacientes, sino que también enriquece el repertorio de los profesionales de la salud, especialmente en enfermería, resultando en una práctica de cuidados de mejor calidad^7^. En este contexto, el uso de terapia con luz de baja intensidad, conocida como *Low-Level Light Therapy* (LLLT), en forma de fotobiomodulación (FBM) y/o fotodinámica (PDT) en el tratamiento adyuvante de LP, se destaca como un ejemplo importante de estos avances^6^. 

La terapia de FBM se caracteriza por el uso de radiación luminosa de baja intensidad, cuyos efectos son desencadenados por la luz en sí y no por el calor. Se compone de radiaciones infrarrojas, visibles, ultravioletas, ionizantes, como los rayos X, y radiaciones gamma. La FBM es radiación electromagnética no ionizante que se diferencia por la longitud de onda, desde el rojo al infrarrojo en el rango de 600-1000 nm, que alcanza diferentes profundidades de la piel, donde estimula funciones celulares promoviendo efectos terapéuticos^8^. La terapia de FBM es reconocida como un enfoque prometedor en el proceso de cicatrización de lesiones, actuando sobre varios mediadores inflamatorios y enzimáticos, modulando estos marcadores y acelerando el proceso de reparación tisular. Como resultado, se observa una reducción en los tiempos de cicatrización, mejor control inflamatorio y, consecuentemente, una disminución en la experiencia del dolor^9-10^. 

Por su parte, la PDT es la combinación de la terapia de FBM con un fotosensibilizador, promoviendo así reacciones fotoquímicas que producen especies reactivas de oxígeno, como el oxígeno singlete, que aplicado en una lesión infectada causa la destrucción de microorganismos, como bacterias y hongos, por medio de daños biológicos irreversibles en la membrana celular^11-12^. 

Considerando la necesidad urgente de fundamentar las prácticas clínicas en datos científicos sólidos, el presente estudio tuvo como objetivo mapear la literatura científica sobre la terapia de fotobiomodulación y fotodinámica en el tratamiento de lesiones por presión. Este enfoque busca no solo agregar nuevos conocimientos al campo, sino también proporcionar datos relevantes para la toma de decisiones clínicas fundamentadas y eficaces. 

## Método

### Tipo de estudio

Se trata de una revisión de alcance que siguió las etapas recomendadas por el *Joanna Briggs Institute* (JBI)^13^ y el *checklist Preferred Reporting Items for Systematic Reviews and Meta-Analyses extension for Scoping Reviews* (PRISMA-ScR)^14^. El registro del protocolo de esta revisión de alcance se encuentra en la plataforma internacional *Open Science Framework* (OSF), DOI: 10.17605/OSF.IO/78NEU.

### Pregunta de investigación

Para determinar la pregunta de investigación, se adoptó la estrategia mnemotécnica Población, Concepto y Contexto (PCC)^13^: Población (P): personas con lesión por presión; Concepto (C): terapia de fotobiomodulación y fotodinámica; Contexto (C): niveles de atención en salud. Así, se definió la siguiente pregunta: ¿Cuáles son las evidencias sobre la terapia de fotobiomodulación y fotodinámica en el proceso de cicatrización de personas con lesión por presión en los niveles de atención en salud? 

### Criterios de elegibilidad

Se incluyeron en esta revisión estudios primarios^15^ cuantitativos, cualitativos y de métodos mixtos, así como todos los tipos de estudios secundarios^15^, tales como revisiones sistemáticas, de alcance, integrativas, narrativas, entre otros, disponibles en su totalidad en los idiomas portugués, inglés y español. El período temporal considerado abarcó los últimos 10 años, de 2014 a 2024, justificado en base a la necesidad de cubrir un período lo suficientemente reciente como para capturar los estudios más actualizados y relevantes sobre el tema en cuestión. Esto permite un análisis exhaustivo de la literatura disponible e incorpora hallazgos recientes que pueden influir en las conclusiones de la investigación.

Se excluyeron artículos que no cumplieron con los criterios establecidos para el objetivo y la pregunta de investigación, así como estudios en modelos animales, *in vitro* y literatura gris.

### Estrategia de búsqueda 

La estrategia de búsqueda fue desarrollada con el apoyo de una bibliotecaria, empleando los operadores booleanos *AND* y *OR* en los idiomas portugués, inglés y español. Se exploraron diversas combinaciones de descriptores obtenidos de los Descriptores en Ciencias de la Salud (DeCS) y *Medical Subject Headings* (MeSH) con el objetivo de garantizar la amplitud y precisión de la búsqueda.

La búsqueda se realizó en el mes de enero de 2024, y se consultaron las siguientes bases de datos: Base de Datos en Enfermería (BDENF), *Cumulative Index to Nursing and Allied Health Literature* (CINAHL), *Cochrane Library*, Embase, Literatura Latinoamericana y del Caribe en Ciencias de la Salud (LILACS), *United States National Library of Medicine/Medical Literature Analysis and Retrieval System Online* (PubMed/MEDLINE), Scopus, *Scientific Electronic Library Online* (SciELO) y *Web of Science* (Figura 1).

**Figura 1:** Estrategia de búsqueda. Florianópolis, SC, Brasil, 2024

Bases de datos	Estrategia de búsqueda
PubMed/MEDLINE*	(“Low-Level Light Therapy” OR “LLLT^†^” OR “Laser Biostimulation” OR “Laser Phototherapy” OR “Low Level Laser” OR “LowLevel Light” OR “Low Power Laser” OR “Low-Level Laser” OR “Low-Level Light” OR “Low-Power Laser” OR Photobiomodula* OR “Laser Therapy” OR “Laser Therapies” OR Photodynamic*) AND (“Wound Healing”[Mesh] OR “Wound Healing” OR “Wound Healings” OR “Cicatrization” OR “Cicatrix” OR “Cicatrization” OR “Scar” OR “Scarring” OR “Scars” OR epithelializ*) AND (“Pressure Ulcer” OR “Bed Sore” OR “Bed Sores” OR Bedsore* OR “Decubitus Ulcer” OR “Decubitus Ulcers” OR “Pressure Sore” OR “Pressure Sores” OR “Pressure Ulcers”) AND (journal article[Publication Type])
Embase (Elsevier)	(“Low-Level Light Therapy” OR “LLLT^†^” OR “Laser Biostimulation” OR “Laser Phototherapy” OR “Low Level Laser” OR “LowLevel Light” OR “Low Power Laser” OR “Low-Level Laser” OR “Low-Level Light” OR “Low-Power Laser” OR Photobiomodula* OR “Laser Therapy” OR “Laser Therapies” OR Photodynamic*) AND (“Wound Healing” OR “Wound Healings” OR “Cicatrization” OR “Cicatrix” OR “Cicatrization” OR “Scar” OR “Scarring” OR “Scars” OR epithelializ*) AND (“Pressure Ulcer” OR “Bed Sore” OR “Bed Sores” OR Bedsore* OR “Decubitus Ulcer” OR “Decubitus Ulcers” OR “Pressure Sore” OR “Pressure Sores” OR “Pressure Ulcers”)
CINAHL^‡^ (EBSCO)	(“Low-Level Light Therapy” OR “LLLT^†^” OR “Laser Biostimulation” OR “Laser Phototherapy” OR “Low Level Laser” OR “LowLevel Light” OR “Low Power Laser” OR “Low-Level Laser” OR “Low-Level Light” OR “Low-Power Laser” OR Photobiomodula* OR “Laser Therapy” OR “Laser Therapies” OR Photodynamic*) AND (“Wound Healing” OR “Wound Healings” OR “Cicatrization” OR “Cicatrix” OR “Cicatrization” OR “Scar” OR “Scarring” OR “Scars” OR epithelializ*) AND (“Pressure Ulcer” OR “Bed Sore” OR “Bed Sores” OR Bedsore* OR “Decubitus Ulcer” OR “Decubitus Ulcers” OR “Pressure Sore” OR “Pressure Sores” OR “Pressure Ulcers”)
Cochrane Library	(“Low-Level Light Therapy” OR “LLLT^†^” OR “Laser Biostimulation” OR “Laser Phototherapy” OR “Low Level Laser” OR “LowLevel Light” OR “Low Power Laser” OR “Low-Level Laser” OR “Low-Level Light” OR “Low-Power Laser” OR Photobiomodula* OR “Laser Therapy” OR “Laser Therapies” OR Photodynamic*) AND (“Wound Healing” OR “Wound Healings” OR “Cicatrization” OR “Cicatrix” OR “Cicatrization” OR “Scar” OR “Scarring” OR “Scars” OR epithelializ*) AND (“Pressure Ulcer” OR “Bed Sore” OR “Bed Sores” OR Bedsore* OR “Decubitus Ulcer” OR “Decubitus Ulcers” OR “Pressure Sore” OR “Pressure Sores” OR “Pressure Ulcers”)
Scopus (Elsevier)	(“Low-Level Light Therapy” OR “LLLT^†^” OR “Laser Biostimulation” OR “Laser Phototherapy” OR “Low Level Laser” OR “LowLevel Light” OR “Low Power Laser” OR “Low-Level Laser” OR “Low-Level Light” OR “Low-Power Laser” OR Photobiomodula* OR “Laser Therapy” OR “Laser Therapies” OR Photodynamic*) AND (“Wound Healing” OR “Wound Healings” OR “Cicatrization” OR “Cicatrix” OR “Cicatrization” OR “Scar” OR “Scarring” OR “Scars” OR epithelializ*) AND (“Pressure Ulcer” OR “Bed Sore” OR “Bed Sores” OR Bedsore* OR “Decubitus Ulcer” OR “Decubitus Ulcers” OR “Pressure Sore” OR “Pressure Sores” OR “Pressure Ulcers”)
Web of Science (Clarivate Analytics)	(“Low-Level Light Therapy” OR “LLLT^†^” OR “Laser Biostimulation” OR “Laser Phototherapy” OR “Low Level Laser” OR “LowLevel Light” OR “Low Power Laser” OR “Low-Level Laser” OR “Low-Level Light” OR “Low-Power Laser” OR Photobiomodula* OR “Laser Therapy” OR “Laser Therapies” OR Photodynamic*) AND (“Wound Healing” OR “Wound Healings” OR “Cicatrization” OR “Cicatrix” OR “Cicatrization” OR “Scar” OR “Scarring” OR “Scars” OR epithelializ*) AND (“Pressure Ulcer” OR “Bed Sore” OR “Bed Sores” OR Bedsore* OR “Decubitus Ulcer” OR “Decubitus Ulcers” OR “Pressure Sore” OR “Pressure Sores” OR “Pressure Ulcers”)
LILACS^§^	(“Terapia com Luz de Baixa Intensidade” OR “Luz de Baixa Intensidade” OR “Bioestimulação a Laser” OR “Laser de Baixa Intensidade” OR “Laser de Baixa Potência” OR Fotobiomodula* OR “Terapia a Laser” OR “laserterapia” OR Fotodinâmic* OR “Terapia por Luz de Baja Intensidad” OR “Luz de Baja Intensidad” OR “Bioestimulación por Láser” OR “Láser de Baja Potencia” OR “Láser de Bajo Poder” OR “Láser de Baja Intensidad” OR “Láser de Bajo Nivel” OR “Terapia por Láser” OR “Low-Level Light Therapy” OR “LLLT^†^” OR “Laser Biostimulation” OR “Laser Phototherapy” OR “Low Level Laser” OR “LowLevel Light” OR “Low Power Laser” OR “Low-Level Laser” OR “Low-Level Light” OR “Low-Power Laser” OR Photobiomodula* OR “Laser Therapy” OR “Laser Therapies” OR Photodynamic*) AND (“Cicatrização” OR “Cicatriz” OR “Cicatrizes” OR Escara* OR epiteliza* OR “Cicatrización de Heridas” OR “Cicatrización” OR “Cicatrices” OR “Wound Healing” OR “Wound Healings” OR “Cicatrization” OR “Cicatrix” OR “Cicatrization” OR “Scar” OR “Scarring” OR “Scars” OR epithelializ*) AND (“Lesão por pressão” OR “Lesão cutânea” OR “Lesão de pele” OR “Úlcera de Decúbito” OR “Úlcera de Pressão” OR “Úlcera por Pressão” OR “Úlceras por Pressão” OR “Úlcera por Presión” OR “Llaga por Presión” OR “Pressure Ulcer” OR “Bed Sore” OR “Bed Sores” OR Bedsore* OR “Decubitus Ulcer” OR “Decubitus Ulcers” OR “Pressure Sore” OR “Pressure Sores” OR “Pressure Ulcers”)
BDENF^||^	(“Terapia com Luz de Baixa Intensidade” OR “Luz de Baixa Intensidade” OR “Bioestimulação a Laser” OR “Laser de Baixa Intensidade” OR “Laser de Baixa Potência” OR Fotobiomodula* OR “Terapia a Laser” OR “laserterapia” OR Fotodinâmic* OR “Terapia por Luz de Baja Intensidad” OR “Luz de Baja Intensidad” OR “Bioestimulación por Láser” OR “Láser de Baja Potencia” OR “Láser de Bajo Poder” OR “Láser de Baja Intensidad” OR “Láser de Bajo Nivel” OR “Terapia por Láser” OR “Low-Level Light Therapy” OR “LLLT^†^” OR “Laser Biostimulation” OR “Laser Phototherapy” OR “Low Level Laser” OR “LowLevel Light” OR “Low Power Laser” OR “Low-Level Laser” OR “Low-Level Light” OR “Low-Power Laser” OR Photobiomodula* OR “Laser Therapy” OR “Laser Therapies” OR Photodynamic*) AND (“Cicatrização” OR “Cicatriz” OR “Cicatrizes” OR Escara* OR epiteliza* OR “Cicatrización de Heridas” OR “Cicatrización” OR “Cicatrices” OR “Wound Healing” OR “Wound Healings” OR “Cicatrization” OR “Cicatrix” OR “Cicatrization” OR “Scar” OR “Scarring” OR “Scars” OR epithelializ*) AND (“Lesão por pressão” OR “Lesão cutânea” OR “Lesão de pele” OR “Úlcera de Decúbito” OR “Úlcera de Pressão” OR “Úlcera por Pressão” OR “Úlceras por Pressão” OR “Úlcera por Presión” OR “Llaga por Presión” OR “Pressure Ulcer” OR “Bed Sore” OR “Bed Sores” OR Bedsore* OR “Decubitus Ulcer” OR “Decubitus Ulcers” OR “Pressure Sore” OR “Pressure Sores” OR “Pressure Ulcers”)
SciELO^¶^	(“Terapia com Luz de Baixa Intensidade” OR “Luz de Baixa Intensidade” OR “Bioestimulação a Laser” OR “Laser de Baixa Intensidade” OR “Laser de Baixa Potência” OR Fotobiomodula* OR “Terapia a Laser” OR “laserterapia” OR Fotodinâmic* OR “Terapia por Luz de Baja Intensidad” OR “Luz de Baja Intensidad” OR “Bioestimulación por Láser” OR “Láser de Baja Potencia” OR “Láser de Bajo Poder” OR “Láser de Baja Intensidad” OR “Láser de Bajo Nivel” OR “Terapia por Láser” OR “Low-Level Light Therapy” OR “LLLT^†^” OR “Laser Biostimulation” OR “Laser Phototherapy” OR “Low Level Laser” OR “Low Level Light” OR “Low Power Laser” OR “Low-Level Laser” OR “Low-Level Light” OR “Low-Power Laser” OR Photobiomodula* OR “Laser Therapy” OR “Laser Therapies” OR Photodynamic*) AND (“Cicatrização” OR “Cicatriz” OR “Cicatrizes” OR Escara* OR epiteliza* OR “Cicatrización de Heridas” OR “Cicatrización” OR “Cicatrices” OR “Wound Healing” OR “Wound Healings” OR “Cicatrization” OR “Cicatrix” OR “Cicatrization” OR “Scar” OR “Scarring” OR “Scars” OR epithelializ*) AND (“Lesão por pressão” OR “Lesão cutânea” OR “Lesão de pele” OR “Úlcera de Decúbito” OR “Úlcera de Pressão” OR “Úlcera por Pressão” OR “Úlceras por Pressão” OR “Úlcera por Presión” OR “Llaga por Presión” OR “Pressure Ulcer” OR “Bed Sore” OR “Bed Sores” OR Bedsore* OR “Decubitus Ulcer” OR “Decubitus Ulcers” OR “Pressure Sore” OR “Pressure Sores” OR “Pressure Ulcers”)

*PubMed/MEDLINE = *United States National Library of Medicine/Medical Literature Analysis and Retrieval System Online*; ^†^LLLT = *Low-level light therapy*; ^‡^CINAHL = *Cumulative Index to Nursing and Allied Health Literature*; ^§^LILACS = Literatura Latinoamericana y del Caribe en Ciencias de la Salud; ^||^BDENF = Base de Datos en Enfermería; ^¶^SciELO = *Scientific Electronic Library Online*

### Selección de los estudios

Las referencias identificadas fueron importadas a Zotero^®^ con el propósito de almacenamiento, organización y detección de estudios duplicados. Se adoptó el *Preferred Reporting Items for Systematic Reviews and Meta-Analyses extension for Scoping Reviews* (PRISMA-ScR)^14^ para guiar tanto el proceso de inclusión como la presentación de los resultados de la selección, siguiendo las cuatro etapas de identificación, cribado, elegibilidad e inclusión. La selección de los estudios se realizó tras la eliminación de duplicados y fue llevada a cabo por dos revisores independientes; cualquier divergencia fue resuelta mediante discusiones internas. La extracción de datos de la muestra final se realizó por medio de una hoja de cálculo elaborada en *Google* ^*®*^ Sheets, lo que permitió una visualización clara de la información obtenida en los estudios seleccionados para la muestra final de la revisión.

### Mapeo y análisis de los datos

Se definió una estrategia de extracción de datos, adaptada conforme al manual del JBI, para seleccionar la siguiente información relevante: 1) caracterización: autor, país, revista, temática, año, título, objetivos y tipo de estudio; 2) aplicabilidad clínica; 3) tipo de tecnología utilizada; 4) principales resultados y limitaciones, información que se organizó en formato de tablas con contenido narrativo en *Microsoft Excel* ^*®*^ .

### Aspectos éticos

Por tratarse de un estudio de revisión de alcance, se dispensa la evaluación por un comité de ética. La presente investigación forma parte del macroproyecto titulado: Evaluación de riesgo y terapia de fotobiomodulación para el tratamiento de LP en personas con condiciones crónicas de salud, el cual cuenta con financiación de la *Fundação de Amparo à Pesquisa e Inovação do Estado de Santa Catarina* (FAPESC), convocatoria pública N°26/2020, término de concesión de apoyo financiero n°2021TR000432.

## Resultados

Se encontraron 157 estudios, de los cuales se excluyeron 99 por duplicidad, cuatro por tratarse de estudios en modelo animal, dos por ser estudios *in vitro* y uno por constituir literatura gris (disertación). Posteriormente, los 51 estudios restantes fueron sometidos al análisis del título y resumen, resultando en la exclusión de veinte por no encuadrarse en el alcance del efecto de la terapia de FBM y PDT en el proceso de cicatrización de personas con lesión por presión, y de siete por no cumplir con el objetivo y la pregunta de investigación establecidos.

Al término de este proceso, quedaron 24 estudios que cumplieron con los criterios de inclusión y fueron seleccionados para integrar esta revisión. Estos estudios se distribuyeron de la siguiente manera en las bases de datos consultadas: PubMed/MEDLINE (n=11), Embase (n=7), CINAHL (n=2), LILACS (n=1), Scopus (n=1), SciELO (n=1) y *Web of Science* (n=1). No se encontraron estudios elegibles en las bases BDENF y *Cochrane Library* tras la exclusión de duplicados. El procedimiento de búsqueda y selección de estudios de esta revisión se encuentra representado en el diagrama de flujo (Figura 2).

**Figura 2:** Diagrama de flujo de búsqueda, conforme a las recomendaciones, adaptado de PRISMA-ScR*. Florianópolis, SC, Brasil, 2024

*PRISMA-ScR = *Preferred Reporting Items for Systematic Reviews and Meta-Analyses extension for Scoping Reviews*

De los 24 estudios incluidos en esta revisión, 8 (33,33%) fueron reportes de caso, 7 (20,17%) revisiones narrativas, 5 (20,83%) revisiones sistemáticas, 1 (4,17%) reporte de experiencia, 1 (4,17%) estudio comparativo aleatorizado, 1 (4,17%) ensayo intervencionista aleatorizado y controlado con placebo, y 1 (4,17%) ensayo clínico aleatorizado. En cuanto al origen, 11 (45,83%) estudios fueron realizados en Brasil, 5 (20,83%) en los Estados Unidos de América (EE. UU.), 2 (8,33%) en Polonia, 2 (8,33%) en India, 1 (4,17%) en Italia, 1 (4,17%) en Irán, 1 (4,17%) el Reino Unido y 1 (4,17%) en España. En relación con el idioma, 19 (73,33%) estudios estaban en inglés y 5 (26,67%) en portugués En cuanto al año de publicación, 1 (4,17%), fue publicado en 2014, 6 (25%), en 2015, 3 (12,5%) en 2017, 2 (8,33%) en 2018, 2 (8,33%) en 2019, 1 (4,17%) en 2020, 2 (8,33%) en 2021, 4 (16,67%) en 2022 y 3 (12,5%) en 2023. La descripción detallada de los estudios, que incluye título, autoría, año, país y revista de publicación, se encuentra en la Figura 3.

**Figura 3:** Caracterización de los estudios incluidos en la revisión de alcance. Florianópolis, SC, Brasil, 2024

Id*	Título	Autoría	Año	País	Periódico
E^†^1^16^	*Lesão por pressão após COVID-19* ^*‡*^ tratada com laserterapia adjuvante: estudo de caso	Lucena, Pinto, Disconzi, Fabris Mazui, Riquinho.	2023	Brazil	*Revista Gaúcha de Enfermagem*
E^†^2^17^	The role of physical therapies in wound healing and assisted scarring	Fernández-Guarino, Bacci, Pérez González, Bermejo-Martínez, Cecilia-Matilla, Hernández-Bule.	2023	Spain	International Journal Of Molecular Sciences
E^†^3^18^	Photobiomodulation therapy and low-level light therapy in wound healing	Aggarwa, Lio.	2023	USA^§^	Lasers in Medical Science
E^†^4^19^	Effectiveness of electrophysical agents for treating pressure injuries: a systematic review	Vieceli, Martins, Hendler, Santos, Neves, Barbosa, et al*.*	2022	Spain	Lasers in Medical Science
E^†^5^20^	Treatment of chronic wounds with methylene blue photodynamic therapy: a case report	Cesar, Winyk, Santo, Queiroz, Soares, Caetano, et al.	2022	Brazil	Photodiagnosis and Photodynamic Therapy
E^†^6^21^	*Laser de baixa intensidade na cicatrização de lesão por pressão estágio 3: relato de experiência*	Sousa, Soares, Borges, Barreto, Caregnato.	2022	Brazil	*Revista Enfermagem Atual in Derme*
E^†^7^22^	Influence of physiotherapy in the vigilant revitalisation of decubitus ulcer: a case report	Bhagdewani, Sasun, Patil.	2022	India	Journal Of Clinical and Diagnostic Research
E^†^8^23^	Phototherapy (cluster multi-diode 630 nm^||^ and 940 nm^||^) on the healing of pressure injury: a pilot study	Baracho, Chaves, Huebner, Oliveira, Ferreira, Lucas.	2021	Brazil	Journal of Vascular Nursing
E^†^9^24^	Eliminating non-healing wounds: a review	Kuffler.	2021	USA^§^	Regenerative Medicine
E^†^10^25^	Skin surface infrared thermography in pressure ulcer outcome prognosis	Bilska, Stangret, Pyzlak, Wojdasiewicz, Szukiewicz.	2020	Poland	Journal of Wound Care
E^†^11^26^	Effect of photobiomodulation on repairing pressure ulcers in adult and elderly patients: a systematic review	Petz, Félix, Roehrs, Pott, Stocco, Marcos.	2019	Poland	Photochemistry and Photobiology
E^†^12^27^	Photobiomodulation therapy for wound care: a potent, noninvasive, photoceutical approach	Mosca, Ong, Albasha, Bass, Arany.	2019	USA^§^	Advances in Skin & Wound Care
E^†^13^28^	*Efeitos da laserterapia no tratamento de lesões por pressão: uma revisão sistemática*	Bernardes, Jurado.	2018	Poland	*Revista Cuidarte*
E^†^14^29^	Effect of laser therapy on expression of angio-and fibrogenic factors, and cytokine concentrations during the healing process of human pressure ulcers	Taradaj, Shay, Dymarek, Sopel, Walewicz, Beeckman, et al.	2018	Poland	International Journal of Medical Sciences
E^†^15^30^	Application of photodynamic therapy, laser therapy, and a cellulose membrane for calcaneal pressure ulcer treatment in a diabetic patient: a case report	Rosa, Silva, Vieira, Tanajura, Gusmão, Oliveira, et al.	2017	Brazil	Photodiagnosis and Photodynamic Therapy
E^†^16^31^	*Efeitos dos lasers Hélio-Neônio (HeNe* ^*¶*^ ) e Arseneto de Gálio (AsGa**) associados à educação em saúde com foco na promoção da saúde de portadores de úlcera por pressão	Fialho, Baron, Brandenburg, Martins.	2017	Brazil	*Revista Médica de Minas Gerais*
E^†^17^32^	Low-level laser therapy in the treatment of pressure ulcers: systematic review	Machado, Viana, Sbruzzi.	2017	Brazil	Lasers in Medical Science
E^†^18^33^	*Laserterapia em úlcera por pressão: avaliação pelas* **Pressure Ulcer Scale for Healing e Nursing Outcomes Classification**	Palagi, Severo, Menegon, Lucena.	2015	Brazil	*Revista da Escola de Enfermagem da USP*
E^†^19^34^	Improving the ability to eliminate wounds and pressure ulcers	Kuffler.	2015	^USA§^	Wound Repair and Regeneration
E^†^20^35^	Photobiomodulation in promoting wound healing: a review	Kuffler.	2015	^USA§^	Regenerative Medicine
E^†^21^36^	Low-Level laser therapy along with intravascular laser in deep pressure ulcer resistant to conventional therapies	Kazemikhoo, Rahbar, Akrami.	2015	Iran	Journal of Skin and Stem Cell
E^†^22^37^	Nonpharmacologic Interventions to heal pressure ulcers in older patients: an overview of systematic reviews (The SENATOR^††^-ONTOP^‡‡^ series)	Pharm, Lozano-Montoya, Abraha, Cherubini, Soiza, O’Mahony, et al.	2015	Italy	Journal of the American Medical Directors Association
E^†^23^38^	Low-level laser therapy as an antimicrobial and antibiofilm technology and its relevance to wound healing	Percival, Francolini, Donelli.	2015	United Kingdom	Future Microbiology
E^†^24^39^	Closure of chronic non healing ankle ulcer with low level laser therapy in a patient presenting with thalassemia intermedia: Case report	Dixit, Agrawal, Sharma, Singh.	2014	India	Indian Journal of Plastic Surgery

*Id = Identificación; ^†^E = Estudio; ^‡^COVID-19 = *Corona Virus Disease* 2019; ^§^EE. UU. = Estados Unidos de América; ^||^nm = Nanómetro; ^¶^HeNe = Helio-neón; **AsGa = Arseniuro de galio; ^††^SENATOR = *Software ENgine for the Assessment & Optimization of drug and non-drug Therapy in Older peRsons*; ^‡‡^ONTOP =*Optimal Evidence-Based Non-drug Therapies in Older People*

La Figura 4 presenta la caracterización y síntesis de los artículos mapeados e incluidos en esta revisión, con las especificaciones sobre FBM y PDT aplicadas y los principales hallazgos de cada estudio.

**Figura 4:** Caracterización de los estudios mapeados según las terapias de fotobiomodulación (FBM) y fotodinámica (PDT) aplicadas. Florianópolis, SC, Brasil, 2024

Id*	Objetivos	Tipo de estudio	Población o artículos incluidos (n^†^)	Especificaciones sobre FBM^‡^ y/o PDT^§^, y frecuencia de aplicación	Principales hallazgos sobre FBM^‡^ y PDT^§^ en LP^||^
E^¶^1^16^	Reportar el tratamiento con laserterapia adyuvante en paciente con LP^||^ tras COVID-19**	Reporte de caso	(n^†^=1)	LLLT^††^ con longitud de onda de 660 y 808 nm^‡‡^, y 1 J^§§^ de láser rojo y 1 J^§§^ de láser infrarrojo. LLLT^††^ aplicada siete veces.	El resultado de la cicatrización mostró curación por segunda intención y cuatro indicadores clínicos: evolución de la reparación tisular con presencia de tejido de epitelización a partir de la 6ª y 7ª aplicación, cobertura completa de tejido de granulación, disminución del tamaño de la lesión y ausencia de exudado, solo trasudado.
E^¶^2^17^	Resumir el papel de las terapias físicas como tratamientos complementarios en la cicatrización de lesiones.	Revisión narrativa	-	-	En LP^||^, la luz roja mejora la cicatrización con mejores resultados en comparación con el LED^||||^ de 805 nm^‡‡^.
E^¶^3^18^	Resumir las aplicaciones de FBM^‡^ en el campo de la cicatrización de lesiones y especificar los resultados actuales en los parámetros utilizados para el tratamiento.	Revisión narrativa	-	-	Los resultados sugieren que una longitud de onda específica de 658 nm^‡‡^, en el rango de luz roja, podría ser más eficaz en el tratamiento de LP^||^.
E^¶^4^19^	Evaluar la eficacia de FBM^‡^, ultrasonido y agentes electrofísicos de alta frecuencia en la cicatrización de lesiones por presión en adultos y personas mayores.	Revisión sistemática	(n^†^=12)	-	La FBM^‡^ mostró una eficacia similar a otras tecnologías recomendadas en otros estudios en la cicatrización de lesiones por presión. La FBM^‡^ con longitud de onda roja (660 nm^‡‡^) en los estadios 2 y 3 de las lesiones por presión promovió eficazmente la curación en comparación con el tratamiento estándar. Se observó que el uso de FBM^‡^ acelera la reparación tisular en lesiones por presión.
E^¶^5^20^	Investigar la seguridad y eficacia del tratamiento con PDT^§^ con uso de azul de metileno en lesiones con signos de inflamación indicativos de infección.	Reporte de caso	(n^†^=3)	PDT^§^ con azul de metileno en concentración de 10 mg/mL^¶¶^ y fuente de LED^||||^ (660 nm^‡‡^). PDT^§^ aplicada una vez por semana durante tres años.	La reducción de tamaño fue sumamente significativa, de 26,2 cm*** de largo y 9,5 cm*** de ancho (248,9 cm^2†††^) a 5,2 cm*** por 4,0 cm*** (20,8 cm^2†††^). Los signos de infección también disminuyeron con la formación de tejido de granulación adherido, pero frágil, con drenaje serosanguinolento y leve olor.
E^¶^6^21^	Reportar las experiencias de una enfermera especialista en el manejo de un caso clínico de una paciente mayor con LP^||^ estadio 3, sometida a terapia láser de baja intensidad.	Reporte de experiencia	(n^†^=1)	LLLT^††^ con longitud de onda roja (660 nm^‡‡^) e infrarroja (808 nm^‡‡^) con 1 J^§§^/cm²^†††^ de láser rojo e infrarrojo. LLLT^††^ aplicada cada 48 horas durante dos meses.	En la 5ª aplicación del láser, se observó una mejora significativa en la secreción y el eritema perilesional. En dos meses de láser y apósitos, se logró la cicatrización completa de la lesión.
E^¶^7^22^	Determinar cómo los fisioterapeutas manejan a pacientes con paraplejia y lesiones por presión sacras, así como la eficacia de la terapia láser en el tratamiento de lesiones graves y grandes.	Reporte de caso	(n^†^=1)	LLLT^††^ con luz de GaAlAs^‡‡‡^, haz continuo, sin contacto (sin pulsación) en longitud de onda de 658 nm^‡‡^ de láser infrarrojo. LLLT^††^ aplicada cinco veces por semana, de 8 a 15 minutos de irradiación, durante un mes.	El tratamiento de lesiones por presión con laserterapia en longitud de onda de 658 nm^‡‡^ pareció ser exitoso.
E^¶^8^23^	Evaluar la eficacia de un prototipo de fototerapia LED^||||^ en participantes con lesión por presión.	Ensayo intervencionista aleatorizado y controlado con placebo	(n^†^=15)	LLLT^††^ con LED^||||^ de longitud de onda de 630 nm^‡‡^ (rojo) y 940 nm^‡‡^ (infrarrojo), dosis de 6 J^§§^/cm^2†††^ (grupo I) y 8 J^§§^/cm2^†††^ (grupo II). LLLT^††^ aplicada tres veces por semana durante ocho semanas, totalizando 24 sesiones por participante.	El área de las LP^||^ mostró una reducción estadísticamente significativa (p<0,001) en las 24 sesiones en todos los grupos de tratamiento. Los grupos con fototerapia LED^||||^ (I y II) presentaron mayor cicatrización en comparación con el grupo III (placebo). La tasa de cicatrización fue mayor en el grupo II, variando entre 94,5% y 98,7% en la 24ª sesión.
E^¶^9^24^	Examinar la etiología de lesiones que no cicatrizan y diferentes tratamientos para el manejo de lesiones.	Revisión narrativa	-	-	Las longitudes de onda de 633-904 nm^‡‡^ generan una curación más rápida en LP^||^. La irradiación mediante LLLT^††^ también induce curación por su efecto antibacteriano. Aunque 650 nm^‡‡^ es más eficaz, la carga bacteriana disminuye bajo tratamiento con longitudes de onda de 830 y 904 nm^‡‡^, pero aumenta con longitud de onda de 670 nm^‡‡^.
E^¶^10^25^	Evaluar la utilidad de SSIT^§§§^ como herramienta pronóstica en el tratamiento de LP^||^ estadios III y IV, con apósitos de hidrocoloide/hidrogel y 20 exposiciones a LLLT^††^, en comparación con apósitos de hidrocoloide solos, en un grupo de pacientes encamados a largo plazo.	Estudio comparativo aleatorizado	(n^†^=43)	LLLT^††^ con longitud de onda de 808 nm^‡‡^. Grupo I: LP^||^ tratadas con apósitos especializados y terapia láser (cinco veces por semana durante cuatro semanas); Grupo II: LP^||^ tratadas con apósitos especializados sin terapia láser.	En el estudio se observaron tres variantes de cicatrización en LP^||^: cicatrización pura con mínima granulación; cicatrización con hipergranulación; y no cicatrizante. Los análisis de patrones termográficos relacionados con SSIT^§§§^ mostraron su dependencia del curso de la curación. El porcentaje de éxito en la cicatrización de LP^||^ alcanzó el 79,2% en el grupo I en comparación con el 73,7% en el grupo II (p<0,05). La variante dominante de cicatrización en el Grupo I fue la cicatrización pura con mínima granulación, mientras que en el Grupo II se observaron igualmente la cicatrización pura con mínima granulación y la cicatrización con hipergranulación.
E^¶^11^26^	Realizar una revisión sistemática evaluando la eficacia de FBM^‡^ en forma de LLLT^††^ en el tratamiento de lesiones por presión en adultos y personas mayores.	Revisión sistemática	(n^†^=5)	-	La FBM^‡^ en longitud de onda infrarroja mostró eficacia en la cicatrización de la lesión por presión, similar al cuidado estándar presentado en diversos estudios. La FBM^‡^ (658 nm^‡‡^) fue eficaz en la promoción de la curación en comparación con el tratamiento estándar.
E^¶^12^27^	Proporcionar antecedentes y examinar evidencia para la aplicación terapéutica de tratamientos de energía lumínica en la cicatrización de heridas.	Revisión narrativa	-	-	El tratamiento con láser de 658 nm^‡‡^ fue más efectivo (70% de cierre, p< 0,05) en la promoción del cierre de LP^||^. En contraste, los tratamientos con láser de 808 y 940 nm^‡‡^ (cierre del 31% y 30%, respectivamente) no parecieron mejorar significativamente las tasas de curación en comparación con el grupo placebo (cierre del 28%).
E^¶^13^28^	Estudiar la eficacia de la laserterapia en el proceso de cicatrización de lesiones por presión.	Revisión sistemática	(n^†^=11)	-	Dosis de 4 J^§§^/cm^2†††^ con longitud de onda de 658 nm^‡‡^ fueron las más efectivas en el tratamiento de las lesiones por presión.
E^¶^14^29^	Evaluar el efecto de la irradiación láser en diferentes longitudes de onda en la expresión de factores de crecimiento y mediadores inflamatorios en fases específicas del proceso de cicatrización de heridas. Pacientes con diagnóstico de lesión crónica relacionada a LP^||^ (II, III, IV) fueron incluidos en el estudio.	Ensayo clínico aleatorizado	(n^†^=67)	LLLT^††^ con luz de GaAlAs^‡‡‡^. Grupo A - 940 nm^‡‡^; Grupo C - 658 nm^‡‡^; Grupo B - 808 nm^‡‡^; Grupo D - terapia simulada. Tratados con LLLT^††^ y analizados una vez al día, cinco días por semana durante un mes.	Para el grupo C (658 nm^‡‡^), la reducción en la concentración de TNF-α^||||||^ fue más significativa (aproximadamente 75%), mientras que en los otros grupos las reducciones no fueron tan marcadas (alrededor del 50%). Parece que el éxito en la cicatrización de heridas tras irradiación a 658 nm^‡‡^ está asociado a un efecto antiinflamatorio, además de estimular fenómenos como la angiogénesis, proliferación o remodelación de tejidos durante el proceso de cierre de la lesión.
E^¶^15^30^	Presentar un caso de LP^||^ en la región del talón en un paciente diabético tratado con una combinación de FBM^‡^, laserterapia y aplicación de membrana de celulosa.	Reporte de caso	(n^†^=1)	LLLT^††^ con láser de 660 nm^‡‡^ (rojo visible) puntual y continuo; y PDT^§^ (placa de 30 LEDs^||||^ de longitud de onda de 450±10 nm^‡‡^ azul visible durante 12 minutos con irradiancia de 30 mW^¶¶¶^/cm^2†††^) y agente fotosensibilizante curcumina al 1,5%. LLLT^††^ aplicada dos veces por semana y una sesión de PDT^§^.	La cicatrización de LP^||^ se completó después de 30 días de tratamiento, observándose la epitelización completa de la lesión.
E^¶^16^31^	Comparar la acción de los láseres HeNe^****^ y AsGa^††††^ en el proceso de cicatrización de LP^||^ y desarrollar un trabajo con medidas preventivas como tratamiento.	Reporte de caso	(n^†^=3)	Caso I - LLLT^††^ con láser HeNe****; Casos II y III - LLLT^††^ con láser AsGa^††††^. Caso I - total de 52 sesiones de LLLT^††^ con HeNe****; Casos II y III - 22 a 46 sesiones de LLLT^††^ con AsGa^††††^.	Paciente 1 - al finalizar las aplicaciones, se observó una reducción de la lesión en un 100% y una mejora significativa en la calidad de vida del usuario. Paciente 2 - cicatrización completa de la lesión en 22 sesiones. Paciente 3 - se redujo la profundidad de la lesión a 0,5 cm***, manteniendo las dimensiones de altura y anchura. Hubo poca mejora en la cicatrización de la lesión, pero una leve mejora en su apariencia.
E^¶^17^32^	Evaluar los efectos de LLLT^††^ en LP^||^ en humanos mediante una revisión sistemática de estudios aleatorizados.	Revisión sistemática	(n^†^=4)	-	Se observaron resultados significativos en el uso de LLLT^††^ con longitud de onda de 658 nm^‡‡^, y no se encontró evidencia para el uso de longitudes de onda superiores a esta en el tratamiento de LP^||^.
E^¶^18^33^	Describir el proceso de cicatrización de una lesión por presión en un paciente crítico tratado con terapia convencional de apósitos y laserterapia de baja intensidad, evaluada mediante PUSH^‡‡‡‡^ y el resultado de cicatrización: segunda intención, de NOC^§§§§^.	Relato de caso	(n^†^=1)	LLLT^††^ con láser AlGaInP^||||||||^, longitud de onda de 660 nm^‡‡^. LLLT^††^ aplicada una vez al día, tres veces por semana, durante cinco semanas consecutivas, totalizando 15 aplicaciones.	Se destaca una reducción en la lesión de 7 cm*** de largo a 1,5 cm*** y de 6 cm*** de ancho a 1,1 cm***, al comparar el primer día y el 15° de evaluación. El tejido epitelial mostraba crecimiento continuo, con reducción significativa de la secreción serosanguinolenta y ausencia de mal olor. Sin embargo, el eritema y la maceración perilesional empeoraron levemente, posiblemente debido a la diuresis en pañales que aumentó la humedad perineal.
E^¶^19^34^	Resumir y evaluar críticamente la evidencia de revisiones sistemáticas sobre intervenciones no farmacológicas para tratar lesiones por presión en pacientes ancianos.	Revisión narrativa	n^†^=110	-	La LLLT^††^ y la iluminación LED^||||^ se emplean en varios tipos de heridas, incluyendo cicatrización de lesiones no cicatrizadas, como pie diabético, lesiones por presión, venosas y posquimioterapia contra lesiones por radiación. En estudios comparativos, la exposición de lesiones crónicas no cicatrizadas a longitudes de onda entre 635 y 810 nm^‡‡^ muestra una curación más rápida.
E^¶^20^35^	Examinar investigaciones sobre la inducción de cicatrización de heridas y presión, activación génica y eliminación del dolor mediante fotobiomodulación y sus mecanismos de acción.	Revisión narrativa	-	-	Pacientes con LP|| grado II en 12 semanas de FBM‡ con pulso monocromático presentan una reducción en el tamaño de la lesión del 80% frente al 50% del grupo control, y la irradiación a 660 y 880 nm^‡‡^ reduce el área inicial de la lesión en 13 veces en comparación con los controles.
E^¶^21^36^	Describir el tratamiento de una lesión crónica por presión en un paciente con lesión medular mediante LLLT^††^.	Reporte de caso	(n^†^=1)	LLLT^††^ con longitud de onda de 980 nm^‡‡^ continua 6 J^§§^/cm2^†††^ para los márgenes y 655 nm^‡‡^ continua 1,8 J^§§/^cm2^†††^ para el lecho de la herida junto con laserterapia intravascular. LLLT^††^ aplicada en días alternos durante 12 sesiones y luego dos veces por semana, totalizando 24 sesiones.	Tras dos sesiones de laserterapia, mejoró la perfusión de la lesión, y al término de la sesión 12 el diámetro de la lesión se redujo a 3×5 cm^2†††^ con una profundidad de 1 cm***. Tras 24 sesiones de LLLT^††^ y cirugía de zetaplastia, la lesión se cicatrizó completamente.
E^¶^22^37^	Resumir y evaluar críticamente la evidencia de revisiones sistemáticas sobre intervenciones no farmacológicas para el tratamiento de LP^||^ en personas mayores.	Revisión sistemática	(n^†^=45)	-	La evidencia es muy baja o insuficiente para respaldar el uso de terapia adyuvante (ultrasonido, presión negativa, láser, electromagnética, luz, ondas de choque, hidroterapia, radiofrecuencia o terapia vibratoria) para mejorar las tasas de cicatrización de LP^||^ en personas mayores
E^¶^23^38^	Identificar literatura que informe únicamente sobre LLLT^††^ sin agentes fotodinámicos, como una tecnología antimicrobiana/antibiofilm y determinar sus efectos en la cicatrización de heridas.	Revisión narrativa	-	-	Los pacientes con LP^||^ tratados con luz infrarroja (956 nm^‡‡^) y roja (637 nm^‡‡^) mostraron una tasa de cicatrización un 49% mayor en comparación con los controles. El tiempo para lograr un 50% y 90% de cierre de la lesión fue significativamente reducido. Tras 5 semanas de tratamiento, el área promedio de la lesión se redujo al 10%, mientras que los controles tardaron 9 semanas en lograrlo. Son pocos los ensayos clínicos con un protocolo estándar para erradicación de infecciones mediante LLLT^††^. Según estudios piloto, la exposición del paciente a luz de 870 nm^‡‡^/930 nm^‡‡^ y dosis de energía superiores a 100 J^§§^/cm^2†††^ parece ser una estrategia terapéutica eficaz.
E^¶^24^39^	Verificar el efecto de LLLT^††^ en forma de LED^||||^ en una lesión crónica que no cicatrizaba en un paciente masculino de 18 años con talasemia.	Reporte de caso	(n^†^=1)	LLLT^††^ en forma de LED^||||^. LLLT^††^ con dosis de 17,3 J^§§^/cm^2†††^ durante 8 minutos durante 2 semanas seguida de dosis proliferativa de 8,65-4,33 J^§§^/cm^2†††^ durante 4 minutos de la semana 3 a la 6.	A la 6ª semana de aplicación, la lesión se cerró.

*Id = Identificación; ^†^n = Número de participantes; ^‡^FBM = Fotobiomodulación; ^§^PDT = Fotodinámica; ^||^LP = Lesión por presión; ^¶^E = Estudio; **COVID-19 = *Corona Virus Disease* 2019; ^††^LLLT = *Low-level light therapy*; ^‡‡^nm = Nanómetro; ^§§^J = *Joule*; ^||||^LED = *Light-emitting diode;* ^¶¶^mg/mL = Miligramos por mililitro; ***cm = Centímetros; ^†††^cm^2^ = Centímetros cuadrados; ^‡‡‡^GaAlAs = Arseniuro de galio-aluminio; ^§§§^SSIT = *Infrared thermography of the skin surface*; ^||||||^TNF-α = *Tumor necrosis factor alpha*; ^¶¶¶^mW = Miliwatt; ****HeNe = Helio-neón; ^††††^AsGa = Arseniuro de galio; ^‡‡‡‡^PUSH *= Pressure Ulcer Scale for Healing*; ^§§§§^NOC *= Nursing Outcomes Classification*; ^||||||||^AlGaInP = Aluminio-galio-indio-fósforo

## Discusión

La terapia con láser de 808 nm resultó en una variante dominante de cicatrización pura (H), mientras que el láser de 658 nm mostró una reducción más significativa en la concentración de TNF-α, sugiriendo un efecto antiinflamatorio y estimulante del proceso de cicatrización^25,29^. Se observa una diversidad de tipos de láser utilizados en LLLT, como se evidencia en la Figura 4. En un estudio de caso^22)^ donde el láser se aplicó cinco veces por semana, entre 8 y 15 minutos de irradiación durante un mes, y en un ensayo clínico aleatorizado^29^ con 67 pacientes tratados y analizados una vez al día, cinco días a la semana durante un mes, se utilizó el láser de Arseniuro de Galio y Aluminio (GaAlAs), un tipo de láser semiconductor introducido alrededor de 1987^40^. En otro caso^33^, con tres aplicaciones por semana totalizando 15 sesiones, se empleó el láser de Aluminio-Galio-Indio-Fósforo (AlGaInP). Además, en otro estudio de caso^31^ con tres pacientes, se usó el láser de Helio-Neón (HeNe), perteneciente a la primera generación de láseres desarrollada entre 1975 y 1985, y el láser de Arseniuro de Galio (AsGa), el primer láser de diodo disponible en el mercado desde 1985^40^.

Asimismo, un ensayo aleatorizado y controlado con placebo^23^ en 15 participantes que recibieron aplicaciones de láser tres veces por semana durante ocho semanas, totalizando 24 sesiones, mostró una reducción estadísticamente significativa en el área de las LPs a lo largo de las sesiones de fototerapia con diodo emisor de luz (LED), destacando el grupo tratado con LED rojo, que presentó la mayor tasa de cicatrización. Estos hallazgos sugieren que diferentes longitudes de onda de láser y LED pueden influir en la cicatrización de LP de distintas maneras, subrayando la importancia de seleccionar el tratamiento adecuado para cada caso clínico.

El período comprendido entre 1995 y 2005, conocido como tercera generación, fue testigo de la introducción del LED, un dispositivo semiconductor con longitudes de onda entre 180 nm y 1 mm. Estos dispositivos, producidos principalmente por emisión espontánea, fueron concebidos como una alternativa económica y fácil de activar electrónicamente^40^. Este tipo también fue empleado en tres casos^20,30,39^, donde se lograron buenos resultados: en uno de ellos la lesión cicatrizó en seis semanas^39^, otro a los 30 días^30^ y el tercero mostró una reducción significativa en el tamaño y mejora en el aspecto de la lesión^20^. En estos dos últimos, se utilizó PDT con fotosensibilizadores como azul de metileno al 1%^20^ y curcumina al 1,5%^30^, resultando en reducción del tamaño de la lesión y formación de tejido de granulación.

Los estudios de caso revisados presentan resultados alentadores en el uso de la laserterapia para el tratamiento de LP. En primer lugar, destaca la eficacia de diversos protocolos de laserterapia en la reducción de lesiones y mejora en la calidad de vida de los pacientes^31^. Además, la laserterapia mostró eficacia en la reducción del tamaño de las lesiones, mejora del tejido epitelial y disminución de los signos de infección^20,22,33^. 

Otro punto común entre los estudios fue la observación de cicatrización completa de las LPs tras un número variable de sesiones de laserterapia, indicando un potencial significativo de esta aproximación terapéutica^21,36,39^. Los resultados también indican que la laserterapia puede promover cicatrización por segunda intención, con reducción del tamaño de la lesión y mejora en los indicadores clínicos, como presencia de tejido epitelial y ausencia de exudado^16,30^. Estos hallazgos sugieren que la laserterapia puede desempeñar un papel importante en la reparación tisular y el manejo de lesiones por presión.

Además de estos hallazgos, es importante señalar que los estudios revisados utilizaron una variedad de longitudes de onda en LLLT. Las longitudes de onda más comunes incluyen 658 nm, 660 nm, 808 nm y 980 nm, cada una con propiedades específicas que pueden influir en los resultados terapéuticos.

Las revisiones narrativas y sistemáticas resaltan la eficacia de diferentes longitudes de onda en LLLT para el tratamiento de LP. La longitud de onda de 658 nm se destaca como una de las más efectivas, mostrando una tasa de cierre significativamente mayor en comparación con otras longitudes de onda, como 808 nm y 940 nm^27^. Además, la luz roja, especialmente en el rango de 633-904 nm, mostró resultados prometedores en la aceleración de la cicatrización de LP^17,24^. La terapia con luz infrarroja, específicamente en 637 nm, también presentó una tasa de cicatrización significativamente mayor en comparación con los grupos control^38^.

Por otro lado, las revisiones sistemáticas destacan la efectividad del láser de 658 nm en dosis específicas, como 4 J/cm^2^, para el tratamiento de LP^28,32^. Asimismo, la FBM con longitud de onda roja (660 nm) en los estadios 2 y 3 de LP^19^ promovió efectivamente la curación en comparación con el tratamiento estándar^19,26^. Sin embargo, la evidencia sobre terapias adyuvantes como la laserterapia para aumentar las tasas de cicatrización de LP en personas mayores es limitada y de calidad muy baja^37^.

Cabe resaltar que, de acuerdo con las revisiones, las longitudes de onda de 650 nm, 660 nm y 880 nm presentaron resultados prometedores en la reducción del tamaño inicial de LP y en la promoción de la cicatrización^18,34-35^. Estos hallazgos refuerzan la importancia de considerar una variedad de longitudes de onda en la terapia con láser para LP, subrayando la necesidad de más investigaciones para desarrollar protocolos terapéuticos estandarizados y guías de tratamiento.

Es importante destacar que el presente estudio buscó mapear la evidencia disponible sobre FBM y PDT en el tratamiento de LP, mostrando resultados prometedores en la cicatrización, reducción del tamaño de las lesiones y mejora de los indicadores clínicos en el tratamiento de LP. Así, los resultados de este estudio pueden contribuir a la formulación de protocolos clínicos, orientando la inversión en innovación tecnológica en salud y, en consecuencia, contribuyendo a la calidad de vida de las personas con LP.

Es imperativo señalar como una limitación de la investigación la escasez de estudios clínicos robustos *in vivo* sobre el fenómeno investigado, lo que resulta en significativas lagunas en cuanto a los parámetros más confiables para el uso de terapias FBM y PDT en el tratamiento de LP. De este modo, es esencial un mayor esfuerzo en investigaciones que puedan establecer guías claras y protocolos estandarizados para orientar la práctica clínica de manera más sólida y maximizar los beneficios terapéuticos de estas terapias.

## Conclusión

En síntesis, los estudios revisados ofrecen una visión amplia sobre el efecto de la FBM y PDT en el tratamiento de LP, destacando resultados prometedores en términos de cicatrización, reducción del tamaño de las lesiones y mejora de los indicadores clínicos. La diversidad de enfoques terapéuticos disponibles, que incluyen diferentes longitudes de onda y protocolos de tratamiento, subraya la importancia de una aproximación personalizada para optimizar los resultados clínicos.

Es importante señalar que, a pesar de los avances alentadores, todavía existen desafíos en la aplicación clínica de estas terapias. La falta de consenso sobre los parámetros ideales de tratamiento, como la selección de la longitud de onda más adecuada y la dosificación óptima de energía, representa una barrera significativa. Además, la heterogeneidad de los estudios y la falta de estandarización en los métodos de evaluación de los resultados dificultan la comparación entre los diferentes ensayos clínicos. Estas cuestiones destacan la necesidad urgente de investigaciones adicionales, así como de colaboración interdisciplinaria entre profesionales de la salud, para mejorar la eficacia y la aplicabilidad clínica de la FBM y PDT en LP.

# Translation: A terapia de fotobiomodulação e fotodinâmica no tratamento de lesão por pressão: revisão de escopo^*^

**DOI:** 10.1590/1518-8345.7495.4489

**Authors:** Alexsandra Martins da Silva, Gabriela Machado Silva, Jerusa Celi Martins, Taline Bavaresco, Maria Elena Echevarría-Guanilo

**Affiliations:** 1 Universidade Federal de Santa Catarina, Florianópolis, SC, Brasil.; 2 Bolsista do Fundo Estadual de Apoio à Manutenção e ao Desenvolvimento da Educação Superior (UNIEDU/FUMDES), Brasil.; 3 Universidade Federal do Rio Grande do Sul, Porto Alegre, RS, Brasil.; 4 Universidade Federal de Santa Catarina, Departamento de Enfermagem, Florianópolis, SC, Brasil.

**Keywords:** Enfermagem, Lesão por Pressão, Terapia a Laser de Baixa Intensidade, Terapia Fotodinâmica, Cuidados de Enfermagem, Revisão.

## Abstract

**Objetivo:**

mapear a literatura científica sobre a terapia de fotobiomodulação e fotodinâmica no tratamento de lesão por pressão.

**Método:**

tratou-se de uma revisão de escopo, conforme preconizado pelo *Joanna Briggs Institute*. Foram incluídos estudos primários e secundários disponíveis na íntegra nos idiomas português, inglês e espanhol, publicados nos últimos 10 anos, de 2014 a 2024, em nove bases de dados.

**Resultados:**

foram incluídos 24 estudos seguindo os critérios de elegibilidade. Os achados sugerem que a fotobiomodulação e fotodinâmica podem desempenhar um papel importante na reparação tecidual, redução do tamanho e melhoria dos indicadores clínicos no manejo de lesão por pressão. Observou-se uma diversidade de tipos de lasers utilizados na fotobiomodulação e os comprimentos de onda comuns mais utilizados incluíram 658 nm, 660 nm, 808 nm e 980 nm.

**Conclusão:**

os estudos identificados mostram que as terapias de fotobiomodulação e fotodinâmica apresentam resultados promissores na cicatrização, redução do tamanho das lesões e melhoria dos indicadores clínicos no tratamento de lesão por pressão.

## Destaques

**(1)** As terapias de luz são eficazes no tratamento de lesões por pressão. **(2)** A cicatrização otimizada é um resultado promissor das terapias estudadas. **(3)** A redução do tamanho das lesões é um efeito positivo das terapias utilizadas. **(4)** Há melhoria clínica apresentada nos indicadores nos estudos revisados.

## Introdução

Segundo o *National Pressure Injury Advisory Panel* (NPIAP), o *European Pressure Ulcer Advisory Panel* (EPUAP) e o *Pan Pacific Pressure Injury Alliance* (PPPIA), a lesão por pressão (LP) é caracterizada como um dano na pele e/ou nos tecidos moles subjacentes, que podem ocorrer em qualquer região do corpo, sobretudo nas áreas que coincidem com proeminências ósseas^1^. 

A aplicação de pressão intensa ou prolongada, juntamente com forças de cisalhamento, pode resultar na formação de lesões que afetam não apenas as camadas superficiais da pele, mas também podem expor estruturas mais profundas como fáscia muscular, tendões e ossos. Fatores adicionais, como estado nutricional, microclima, circulação sanguínea comprometida e comorbidades, podem contribuir para o desenvolvimento e agravamento das LP^1-2^. 

As hospitalizações agudas, especialmente em indivíduos com mobilidade física prejudicada, são consideradas um fator de risco para o desenvolvimento de LP^3^. Além disso, as LP representam um indicador negativo da qualidade da assistência prestada pela equipe de saúde multidisciplinar, sendo consideradas como eventos adversos potencialmente evitáveis^3-5^.

A presença de uma LP desencadeia uma série de eventos biológicos destinados à reparação tecidual, com o intuito de remediar o dano cutâneo. Entretanto, o mecanismo fisiológico de reparação muitas vezes se mostra inadequado, sobretudo em pacientes que apresentam debilidades decorrentes de condições médicas. Nesse sentido, torna-se imprescindível a adoção de medidas adicionais para acelerar o processo de cicatrização e mitigar os riscos de complicações associadas^6^.

Ademais, a integração de tecnologias na área da saúde, como no tratamento de lesões, não só impulsiona avanços significativos na qualidade de vida dos pacientes, mas também enriquece o repertório dos profissionais da saúde, sobretudo da enfermagem, resultando em uma prática de cuidados de melhor qualidade^7^. Nesse contexto, o uso de terapia com luz de baixa intensidade, conhecida como *Low-Level Light Therapy* (LLLT), na forma de fotobiomodulação (FBM) e/ou fotodinâmica (PDT) no tratamento adjuvante de LP, destaca-se como um exemplo importante desses avanços^6^. 

A terapia de FBM é caracterizada pelo uso de radiação luminosa de baixa intensidade, cujos efeitos são desencadeados pela luz em si e não pelo calor. É formada pelas radiações infravermelhas, visíveis, ultravioletas, ionizantes, como raio X, e radiações gama. A FBM são radiações eletromagnéticas não ionizantes que se diferenciam pelo comprimento de onda, de vermelho ao infravermelho na faixa entre 600-1000 nm, que atingem diferentes profundidades da pele, onde estimulam funções celulares promovendo efeitos terapêuticos^8^. A terapia de FBM é reconhecida como uma abordagem promissora no processo de cicatrização de lesões, atuando em vários mediadores inflamatórios e enzimáticos, modulando esses marcadores e acelerando o processo de reparação tecidual. Como resultado, observa-se uma redução nos tempos de cicatrização, melhor controle inflamatório e, consequentemente, uma diminuição na experiência de dor^9-10^. 

Por sua vez, a PDT é a combinação da terapia de FBM com um fotossensibilizador, assim, ela promove reações fotoquímicas que produzem espécies reativas de oxigênio, como o oxigênio singlete, que aplicado em uma lesão infectada causa a destruição de microrganismos, como bactérias e fungos, por meio de danos biológicos irreversíveis à membrana celular^11-12^. 

Ao considerar a necessidade premente de embasar as práticas clínicas em dados científicos sólidos, o presente estudo teve como objetivo mapear a literatura científica sobre a terapia de fotobiomodulação e fotodinâmica no tratamento de lesão por pressão. Essa abordagem busca não apenas agregar novos conhecimentos ao campo, mas também fornecer subsídios relevantes para a tomada de decisões clínicas fundamentadas e eficazes. 

## Método

### Tipo de estudo

Trata-se de uma revisão de escopo que seguiu as etapas recomendadas pelo *Joanna Briggs Institute* (JBI)^13^ e do *checklist Preferred Reporting Items for Systematic reviews and Meta-Analyses extension for Scoping Reviews* (PRISMA-ScR)^14^. O registro do protocolo desta revisão de escopo consta na plataforma internacional *Open Science Framework* (OSF), DOI: 10.17605/OSF.IO/78NEU. 

### Questão de pesquisa

Para determinar a questão de pesquisa, adotou-se a estratégia mnemônica População, Conceito e Contexto (PCC)^13^. População (P): refere-se à pessoa com lesão por pressão; Conceito (C): terapia de fotobiomodulação e fotodinâmica; Contexto (C): níveis de atenção à saúde. Assim, definiu-se a seguinte questão: quais são as evidências sobre terapia de fotobiomodulação e fotodinâmica no processo de cicatrização de pessoas com lesão por pressão nos níveis de atenção à saúde? 

### Critérios de elegibilidade

Foram incluídos nesta revisão estudos primários^15^ quantitativos, qualitativos e de métodos mistos, assim como todos os tipos de estudos secundários^15^, tais como revisões sistemáticas, de escopo, integrativas, narrativas, entre outros, disponíveis na íntegra nos idiomas português, inglês e espanhol. O período temporal considerado abrangeu os últimos 10 anos, de 2014 a 2024, justificado com base na necessidade de abranger um período recente o suficiente para capturar os estudos mais atualizados e relevantes sobre o tema em questão. Isso permite uma análise abrangente da literatura disponível e incorpora descobertas recentes que podem influenciar as conclusões da pesquisa. 

Foram excluídos artigos que não atenderam aos critérios estabelecidos para o objetivo e questão de pesquisa, assim como estudos em modelo animal, *in vitro* e literatura cinzenta.

### Estratégia de busca 

A estratégia de busca foi desenvolvida com o suporte de uma bibliotecária, empregando os operadores booleanos *AND* e *OR* nos idiomas português, inglês e espanhol. Foram exploradas diversas combinações de descritores obtidos dos Descritores em Ciências da Saúde (DeCS) e *Medical Subject Headings* (MeSH) com o intuito de garantir a amplitude e a precisão da pesquisa. 

A busca ocorreu no mês de janeiro de 2024 e foram consultadas as seguintes bases de dados: Base de Dados em Enfermagem (BDENF), *Cumulative Index to Nursing and Allied Health Literature* (CINAHL), *Cochrane Library*, Embase, Literatura Latino-Americana e do Caribe em Ciências da Saúde (LILACS), *United States National Library of Medicine/Medical Literature Analysis and Retrieval System Online* (PubMed/MEDLINE), Scopus, *Scientific Electronic Library Online* (SciELO) e *Web of Science* (Figura 1). 

**Figura 1:** Estratégia de busca. Florianópolis, SC, Brasil, 2024

Base de dados	Estratégia de busca
PubMed/MEDLINE*	(“Low-Level Light Therapy” OR “LLLT^†^” OR “Laser Biostimulation” OR “Laser Phototherapy” OR “Low Level Laser” OR “LowLevel Light” OR “Low Power Laser” OR “Low-Level Laser” OR “Low-Level Light” OR “Low-Power Laser” OR Photobiomodula* OR “Laser Therapy” OR “Laser Therapies” OR Photodynamic*) AND (“Wound Healing”[Mesh] OR “Wound Healing” OR “Wound Healings” OR “Cicatrization” OR “Cicatrix” OR “Cicatrization” OR “Scar” OR “Scarring” OR “Scars” OR epithelializ*) AND (“Pressure Ulcer” OR “Bed Sore” OR “Bed Sores” OR Bedsore* OR “Decubitus Ulcer” OR “Decubitus Ulcers” OR “Pressure Sore” OR “Pressure Sores” OR “Pressure Ulcers”) AND (journal article[Publication Type])
Embase (Elsevier)	(“Low-Level Light Therapy” OR “LLLT^†^” OR “Laser Biostimulation” OR “Laser Phototherapy” OR “Low Level Laser” OR “LowLevel Light” OR “Low Power Laser” OR “Low-Level Laser” OR “Low-Level Light” OR “Low-Power Laser” OR Photobiomodula* OR “Laser Therapy” OR “Laser Therapies” OR Photodynamic*) AND (“Wound Healing” OR “Wound Healings” OR “Cicatrization” OR “Cicatrix” OR “Cicatrization” OR “Scar” OR “Scarring” OR “Scars” OR epithelializ*) AND (“Pressure Ulcer” OR “Bed Sore” OR “Bed Sores” OR Bedsore* OR “Decubitus Ulcer” OR “Decubitus Ulcers” OR “Pressure Sore” OR “Pressure Sores” OR “Pressure Ulcers”)
CINAHL^‡^ (EBSCO)	(“Low-Level Light Therapy” OR “LLLT^†^” OR “Laser Biostimulation” OR “Laser Phototherapy” OR “Low Level Laser” OR “LowLevel Light” OR “Low Power Laser” OR “Low-Level Laser” OR “Low-Level Light” OR “Low-Power Laser” OR Photobiomodula* OR “Laser Therapy” OR “Laser Therapies” OR Photodynamic*) AND (“Wound Healing” OR “Wound Healings” OR “Cicatrization” OR “Cicatrix” OR “Cicatrization” OR “Scar” OR “Scarring” OR “Scars” OR epithelializ*) AND (“Pressure Ulcer” OR “Bed Sore” OR “Bed Sores” OR Bedsore* OR “Decubitus Ulcer” OR “Decubitus Ulcers” OR “Pressure Sore” OR “Pressure Sores” OR “Pressure Ulcers”)
Cochrane Library	(“Low-Level Light Therapy” OR “LLLT^†^” OR “Laser Biostimulation” OR “Laser Phototherapy” OR “Low Level Laser” OR “LowLevel Light” OR “Low Power Laser” OR “Low-Level Laser” OR “Low-Level Light” OR “Low-Power Laser” OR Photobiomodula* OR “Laser Therapy” OR “Laser Therapies” OR Photodynamic*) AND (“Wound Healing” OR “Wound Healings” OR “Cicatrization” OR “Cicatrix” OR “Cicatrization” OR “Scar” OR “Scarring” OR “Scars” OR epithelializ*) AND (“Pressure Ulcer” OR “Bed Sore” OR “Bed Sores” OR Bedsore* OR “Decubitus Ulcer” OR “Decubitus Ulcers” OR “Pressure Sore” OR “Pressure Sores” OR “Pressure Ulcers”)
Scopus (Elsevier)	(“Low-Level Light Therapy” OR “LLLT^†^” OR “Laser Biostimulation” OR “Laser Phototherapy” OR “Low Level Laser” OR “LowLevel Light” OR “Low Power Laser” OR “Low-Level Laser” OR “Low-Level Light” OR “Low-Power Laser” OR Photobiomodula* OR “Laser Therapy” OR “Laser Therapies” OR Photodynamic*) AND (“Wound Healing” OR “Wound Healings” OR “Cicatrization” OR “Cicatrix” OR “Cicatrization” OR “Scar” OR “Scarring” OR “Scars” OR epithelializ*) AND (“Pressure Ulcer” OR “Bed Sore” OR “Bed Sores” OR Bedsore* OR “Decubitus Ulcer” OR “Decubitus Ulcers” OR “Pressure Sore” OR “Pressure Sores” OR “Pressure Ulcers”)
Web of Science (Clarivate Analytics)	(“Low-Level Light Therapy” OR “LLLT^†^” OR “Laser Biostimulation” OR “Laser Phototherapy” OR “Low Level Laser” OR “LowLevel Light” OR “Low Power Laser” OR “Low-Level Laser” OR “Low-Level Light” OR “Low-Power Laser” OR Photobiomodula* OR “Laser Therapy” OR “Laser Therapies” OR Photodynamic*) AND (“Wound Healing” OR “Wound Healings” OR “Cicatrization” OR “Cicatrix” OR “Cicatrization” OR “Scar” OR “Scarring” OR “Scars” OR epithelializ*) AND (“Pressure Ulcer” OR “Bed Sore” OR “Bed Sores” OR Bedsore* OR “Decubitus Ulcer” OR “Decubitus Ulcers” OR “Pressure Sore” OR “Pressure Sores” OR “Pressure Ulcers”)
LILACS^§^	(“Terapia com Luz de Baixa Intensidade” OR “Luz de Baixa Intensidade” OR “Bioestimulação a Laser” OR “Laser de Baixa Intensidade” OR “Laser de Baixa Potência” OR Fotobiomodula* OR “Terapia a Laser” OR “laserterapia” OR Fotodinâmic* OR “Terapia por Luz de Baja Intensidad” OR “Luz de Baja Intensidad” OR “Bioestimulación por Láser” OR “Láser de Baja Potencia” OR “Láser de Bajo Poder” OR “Láser de Baja Intensidad” OR “Láser de Bajo Nivel” OR “Terapia por Láser” OR “Low-Level Light Therapy” OR “LLLT^†^” OR “Laser Biostimulation” OR “Laser Phototherapy” OR “Low Level Laser” OR “LowLevel Light” OR “Low Power Laser” OR “Low-Level Laser” OR “Low-Level Light” OR “Low-Power Laser” OR Photobiomodula* OR “Laser Therapy” OR “Laser Therapies” OR Photodynamic*) AND (“Cicatrização” OR “Cicatriz” OR “Cicatrizes” OR Escara* OR epiteliza* OR “Cicatrización de Heridas” OR “Cicatrización” OR “Cicatrices” OR “Wound Healing” OR “Wound Healings” OR “Cicatrization” OR “Cicatrix” OR “Cicatrization” OR “Scar” OR “Scarring” OR “Scars” OR epithelializ*) AND (“Lesão por pressão” OR “Lesão cutânea” OR “Lesão de pele” OR “Úlcera de Decúbito” OR “Úlcera de Pressão” OR “Úlcera por Pressão” OR “Úlceras por Pressão” OR “Úlcera por Presión” OR “Llaga por Presión” OR “Pressure Ulcer” OR “Bed Sore” OR “Bed Sores” OR Bedsore* OR “Decubitus Ulcer” OR “Decubitus Ulcers” OR “Pressure Sore” OR “Pressure Sores” OR “Pressure Ulcers”)
BDENF^||^	(“Terapia com Luz de Baixa Intensidade” OR “Luz de Baixa Intensidade” OR “Bioestimulação a Laser” OR “Laser de Baixa Intensidade” OR “Laser de Baixa Potência” OR Fotobiomodula* OR “Terapia a Laser” OR “laserterapia” OR Fotodinâmic* OR “Terapia por Luz de Baja Intensidad” OR “Luz de Baja Intensidad” OR “Bioestimulación por Láser” OR “Láser de Baja Potencia” OR “Láser de Bajo Poder” OR “Láser de Baja Intensidad” OR “Láser de Bajo Nivel” OR “Terapia por Láser” OR “Low-Level Light Therapy” OR “LLLT^†^” OR “Laser Biostimulation” OR “Laser Phototherapy” OR “Low Level Laser” OR “LowLevel Light” OR “Low Power Laser” OR “Low-Level Laser” OR “Low-Level Light” OR “Low-Power Laser” OR Photobiomodula* OR “Laser Therapy” OR “Laser Therapies” OR Photodynamic*) AND (“Cicatrização” OR “Cicatriz” OR “Cicatrizes” OR Escara* OR epiteliza* OR “Cicatrización de Heridas” OR “Cicatrización” OR “Cicatrices” OR “Wound Healing” OR “Wound Healings” OR “Cicatrization” OR “Cicatrix” OR “Cicatrization” OR “Scar” OR “Scarring” OR “Scars” OR epithelializ*) AND (“Lesão por pressão” OR “Lesão cutânea” OR “Lesão de pele” OR “Úlcera de Decúbito” OR “Úlcera de Pressão” OR “Úlcera por Pressão” OR “Úlceras por Pressão” OR “Úlcera por Presión” OR “Llaga por Presión” OR “Pressure Ulcer” OR “Bed Sore” OR “Bed Sores” OR Bedsore* OR “Decubitus Ulcer” OR “Decubitus Ulcers” OR “Pressure Sore” OR “Pressure Sores” OR “Pressure Ulcers”)
SciELO^¶^	(“Terapia com Luz de Baixa Intensidade” OR “Luz de Baixa Intensidade” OR “Bioestimulação a Laser” OR “Laser de Baixa Intensidade” OR “Laser de Baixa Potência” OR Fotobiomodula* OR “Terapia a Laser” OR “laserterapia” OR Fotodinâmic* OR “Terapia por Luz de Baja Intensidad” OR “Luz de Baja Intensidad” OR “Bioestimulación por Láser” OR “Láser de Baja Potencia” OR “Láser de Bajo Poder” OR “Láser de Baja Intensidad” OR “Láser de Bajo Nivel” OR “Terapia por Láser” OR “Low-Level Light Therapy” OR “LLLT^†^” OR “Laser Biostimulation” OR “Laser Phototherapy” OR “Low Level Laser” OR “Low Level Light” OR “Low Power Laser” OR “Low-Level Laser” OR “Low-Level Light” OR “Low-Power Laser” OR Photobiomodula* OR “Laser Therapy” OR “Laser Therapies” OR Photodynamic*) AND (“Cicatrização” OR “Cicatriz” OR “Cicatrizes” OR Escara* OR epiteliza* OR “Cicatrización de Heridas” OR “Cicatrización” OR “Cicatrices” OR “Wound Healing” OR “Wound Healings” OR “Cicatrization” OR “Cicatrix” OR “Cicatrization” OR “Scar” OR “Scarring” OR “Scars” OR epithelializ*) AND (“Lesão por pressão” OR “Lesão cutânea” OR “Lesão de pele” OR “Úlcera de Decúbito” OR “Úlcera de Pressão” OR “Úlcera por Pressão” OR “Úlceras por Pressão” OR “Úlcera por Presión” OR “Llaga por Presión” OR “Pressure Ulcer” OR “Bed Sore” OR “Bed Sores” OR Bedsore* OR “Decubitus Ulcer” OR “Decubitus Ulcers” OR “Pressure Sore” OR “Pressure Sores” OR “Pressure Ulcers”)

*PubMed/MEDLINE = *United States National Library of Medicine/Medical Literature Analysis and Retrieval System Online*; ^†^LLLT = *Low-level light therapy*; ^‡^CINAHL = *Cumulative Index to Nursing and Allied Health Literature*; ^§^LILACS = Literatura Latino-Americana e do Caribe em Ciências da Saúde; ^||^BDENF = Base de Dados em Enfermagem; ^¶^SciELO = *Scientific Electronic Library Online*

### Seleção dos estudos

As referências identificadas foram importadas para o Zotero^®^ com o propósito de armazenamento, organização e detecção de estudos duplicados. O *Preferred Reporting Items for Systematic Reviews and Meta-Analyses extension for Scoping Reviews* (PRISMA-ScR)^14^ foi adotado para guiar tanto o processo de inclusão quanto a apresentação dos resultados da seleção, seguindo as quatro etapas de identificação, triagem, elegibilidade e inclusão. A seleção dos estudos foi realizada após a remoção de duplicatas e conduzida por dois revisores independentes, e quaisquer divergências foram resolvidas por meio de discussões internas. A extração dos dados da amostra final foi realizada por meio de uma planilha elaborada no *Google* ^*®*^ Planilhas, o que possibilitou uma visualização clara das informações obtidas nos estudos selecionados para a amostra final da revisão.

### Mapeamento e análise dos dados

Uma estratégia de extração de dados foi definida e adaptada de acordo o manual do JBI, a fim de selecionarem-se as seguintes informações relevantes: 1) caracterização: autor, país, periódico, temática, ano, título, objetivos e tipo de estudo; 2) aplicabilidade clínica; 3) tipo de tecnologia utilizada; 4) principais resultados e limitações, informações essas que foram organizadas no formato de tabelas com conteúdo narrativo no Microsoft Excel^®^.

### Aspectos éticos

Por se tratar de um estudo de revisão de escopo, dispensa-se avaliação de comitê de ética. A presente pesquisa integra o macroprojeto intitulado: “Avaliação de risco e terapia de fotobiomodulação para tratamento de LP de pessoas em condições crônicas de saúde”, o qual conta com financiamento da Fundação de Amparo à Pesquisa e Inovação do Estado de Santa Catarina (FAPESC) chamada pública N°26/2020, termo de outorga de apoio financeiro n°2021TR000432.

## Resultados

Foram encontrados 157 estudos, dos quais 99 foram excluídos devido à duplicidade, quatro por se tratarem de estudos em modelo animal, dois por serem estudos *in vitro* e um por constituir-se em literatura cinzenta (dissertação). Posteriormente, 51 estudos restantes foram submetidos à análise do título e resumo, resultando na exclusão de vinte por não se enquadrarem no escopo do efeito da terapia de FBM e PDT no processo de cicatrização de pessoas com lesão por pressão, e de sete por não atenderem ao objetivo e à pergunta de pesquisa estabelecidos. 

Ao término deste processo, restaram 24 estudos que preencheram os critérios de inclusão e foram selecionados para integrar esta revisão. Esses estudos foram distribuídos da seguinte maneira nas bases de dados consultadas: PubMed/MEDLINE (n=11), Embase (n=7), CINAHL (n=2), LILACS (n=1), Scopus (n=1), SciELO (n=1) e *Web of Science* (n=1). Não foram encontrados estudos elegíveis nas bases BDENF e *Cochrane Library* após a exclusão das duplicidades. O procedimento de busca e seleção dos estudos desta revisão está devidamente representado no fluxograma (Figura 2).

**Figura 2:** Fluxograma de buscas, conforme recomendações, adaptado do PRISMA-ScR*. Florianópolis, SC, Brasil, 2024

*PRISMA-ScR = *Preferred Reporting Items for Systematic Reviews and Meta-Analyses extension for Scoping Reviews*

Dos 24 estudos incluídos nesta revisão, 8 (33,33%) eram relatos de caso, 7 (20,17%) revisões narrativas, 5 (20,83%) revisões sistemáticas, 1 (4,17%) relato de experiência,1 (4,17%) estudo comparativo aleatorizado, 1 (4,17%) ensaio intervencionista randomizado e controlado por placebo e 1 (4,17%) ensaio clínico randomizado. Quanto à origem, 11 (45,83%), foram conduzidos no Brasil, 5 (20,83%), nos Estados Unidos da América (EUA), 2 (8,33%) na Polônia, 2 (8,33%) na Índia (8,33%), 1 (4,17%) na Itália, 1 (4,17%) no Irã, 1 (4,17%) no Reino Unido e 1 (4,17%) na Espanha. Em relação ao idioma, 19 (73,33%) estudos estavam em inglês e 5 (26,67%) em português. Quanto ao ano de publicação, 1 (4,17%) foi publicado em 2014, 6 (25%) em 2015, 3 (12,5%) em 2017, 2 (8,33%) em 2018, 2 (8,33%) em 2019, 1 (4,17%) em 2020, 2 (8,33%) em 2021, 4 (16,67%) em 2022, e 3 (12,5%) em 2023. A descrição detalhada dos estudos, contendo título, autoria, ano, país e periódico de publicação, encontra-se na Figura 3. 

**Figura 3:** Caracterização dos estudos incluídos na revisão de escopo. Florianópolis, SC, Brasil, 2024

Id*	Título	Autoria	Ano	País	Periódico
E^†^1^16^	*Lesão por pressão após COVID-19* ^*‡*^ tratada com laserterapia adjuvante: estudo de caso	Lucena, Pinto, Disconzi, Fabris Mazui, Riquinho.	2023	Brazil	*Revista Gaúcha de Enfermagem*
E^†^2^17^	The role of physical therapies in wound healing and assisted scarring	Fernández-Guarino, Bacci, Pérez González, Bermejo-Martínez, Cecilia-Matilla, Hernández-Bule.	2023	Spain	International Journal Of Molecular Sciences
E^†^3^18^	Photobiomodulation therapy and low-level light therapy in wound healing	Aggarwa, Lio.	2023	USA^§^	Lasers in Medical Science
E^†^4^19^	Effectiveness of electrophysical agents for treating pressure injuries: a systematic review	Vieceli, Martins, Hendler, Santos, Neves, Barbosa, et al*.*	2022	Spain	Lasers in Medical Science
E^†^5^20^	Treatment of chronic wounds with methylene blue photodynamic therapy: a case report	Cesar, Winyk, Santo, Queiroz, Soares, Caetano, et al.	2022	Brazil	Photodiagnosis and Photodynamic Therapy
E^†^6^21^	*Laser de baixa intensidade na cicatrização de lesão por pressão estágio 3: relato de experiência*	Sousa, Soares, Borges, Barreto, Caregnato.	2022	Brazil	*Revista Enfermagem Atual in Derme*
E^†^7^22^	Influence of physiotherapy in the vigilant revitalisation of decubitus ulcer: a case report	Bhagdewani, Sasun, Patil.	2022	India	Journal Of Clinical and Diagnostic Research
E^†^8^23^	Phototherapy (cluster multi-diode 630 nm^||^ and 940 nm^||^) on the healing of pressure injury: a pilot study	Baracho, Chaves, Huebner, Oliveira, Ferreira, Lucas.	2021	Brazil	Journal of Vascular Nursing
E^†^9^24^	Eliminating non-healing wounds: a review	Kuffler.	2021	USA^§^	Regenerative Medicine
E^†^10^25^	Skin surface infrared thermography in pressure ulcer outcome prognosis	Bilska, Stangret, Pyzlak, Wojdasiewicz, Szukiewicz.	2020	Poland	Journal of Wound Care
E^†^11^26^	Effect of photobiomodulation on repairing pressure ulcers in adult and elderly patients: a systematic review	Petz, Félix, Roehrs, Pott, Stocco, Marcos.	2019	Poland	Photochemistry and Photobiology
E^†^12^27^	Photobiomodulation therapy for wound care: a potent, noninvasive, photoceutical approach	Mosca, Ong, Albasha, Bass, Arany.	2019	USA^§^	Advances in Skin & Wound Care
E^†^13^28^	*Efeitos da laserterapia no tratamento de lesões por pressão: uma revisão sistemática*	Bernardes, Jurado.	2018	Poland	*Revista Cuidarte*
E^†^14^29^	Effect of laser therapy on expression of angio-and fibrogenic factors, and cytokine concentrations during the healing process of human pressure ulcers	Taradaj, Shay, Dymarek, Sopel, Walewicz, Beeckman, et al.	2018	Poland	International Journal of Medical Sciences
E^†^15^30^	Application of photodynamic therapy, laser therapy, and a cellulose membrane for calcaneal pressure ulcer treatment in a diabetic patient: a case report	Rosa, Silva, Vieira, Tanajura, Gusmão, Oliveira, et al.	2017	Brazil	Photodiagnosis and Photodynamic Therapy
E^†^16^31^	*Efeitos dos lasers Hélio-Neônio (HeNe* ^*¶*^ ) e Arseneto de Gálio (AsGa**) associados à educação em saúde com foco na promoção da saúde de portadores de úlcera por pressão	Fialho, Baron, Brandenburg, Martins.	2017	Brazil	*Revista Médica de Minas Gerais*
E^†^17^32^	Low-level laser therapy in the treatment of pressure ulcers: systematic review	Machado, Viana, Sbruzzi.	2017	Brazil	Lasers in Medical Science
E^†^18^33^	*Laserterapia em úlcera por pressão: avaliação pelas* **Pressure Ulcer Scale for Healing e Nursing Outcomes Classification**	Palagi, Severo, Menegon, Lucena.	2015	Brazil	*Revista da Escola de Enfermagem da USP*
E^†^19^34^	Improving the ability to eliminate wounds and pressure ulcers	Kuffler.	2015	^USA§^	Wound Repair and Regeneration
E^†^20^35^	Photobiomodulation in promoting wound healing: a review	Kuffler.	2015	^USA§^	Regenerative Medicine
E^†^21^36^	Low-Level laser therapy along with intravascular laser in deep pressure ulcer resistant to conventional therapies	Kazemikhoo, Rahbar, Akrami.	2015	Iran	Journal of Skin and Stem Cell
E^†^22^37^	Nonpharmacologic Interventions to heal pressure ulcers in older patients: an overview of systematic reviews (The SENATOR^††^-ONTOP^‡‡^ series)	Pharm, Lozano-Montoya, Abraha, Cherubini, Soiza, O’Mahony, et al.	2015	Italy	Journal of the American Medical Directors Association
E^†^23^38^	Low-level laser therapy as an antimicrobial and antibiofilm technology and its relevance to wound healing	Percival, Francolini, Donelli.	2015	United Kingdom	Future Microbiology
E^†^24^39^	Closure of chronic non healing ankle ulcer with low level laser therapy in a patient presenting with thalassemia intermedia: Case report	Dixit, Agrawal, Sharma, Singh.	2014	India	Indian Journal of Plastic Surgery

*Id = Identificação; ^†^E = Estudo; ^‡^COVID-19 = *Corona Virus Disease* 2019; ^§^EUA = Estados Unidos da América; ^||^nm = Nanômetro; ^¶^HeNe = Hélio-neônio; **AsGa = Arseneto de gálio; ^††^SENATOR = *Software ENgine for the Assessment & Optimization of drug and non-drug Therapy in Older peRsons*; ^‡‡^ONTOP =*Optimal Evidence-Based Non-drug Therapies in Older People*

A Figura 4 traz a caracterização e síntese dos artigos mapeados e incluídos nesta revisão com as especificações sobre FBM e PDT aplicadas e aos principais achados de cada estudo.

**Figura 4:** Caracterização dos estudos mapeados segundo as terapias fotobiomodulação (FBM) e fotodinâmica (PDT) aplicadas. Florianópolis, SC, Brasil, 2024

Id*	Objetivos	Tipo de estudo	População ou artigos incluídos (n^†^)	Especificações sobre FBM^‡^ e/ou PDT^§^, e frequência de aplicação	Principais achados sobre FBM^‡^ e PDT^§^ em LP^||^
E^¶^1^16^	Relatar o tratamento por laserterapia adjuvante em paciente com LP^||^ após COVID-19**	Relato de caso	(n^†^=1)	LLLT^††^ com comprimento de onda de 660 e 808 nm^‡‡^, e 1 J^§§^ de laser vermelho e 1 J^§§^ de laser infravermelho. LLLT^††^ aplicada sete vezes.	O resultado da cicatrização das lesões apresentou cicatrização por segunda intenção e quatro indicadores clínicos: evolução da reparação tecidual com presença de tecido de epitelização a partir da 6º e 7º de aplicação, completamente recoberta granulação, tamanho da lesão diminuído, e ausência de exsudato, apenas transudato.
E^¶^2^17^	Resumir o papel das terapias físicas como tratamentos complementares na cicatrização de lesões.	Revisão narrativa	-	-	Em LP^||^, a luz vermelha aumenta a cicatrização com melhores resultados quando comparada com a LED^||||^ 805 nm^‡‡^.
E^¶^3^18^	Resumir as aplicações da FBM^‡^ no campo da cicatrização de lesões e especificar os resultados atuais nos parâmetros utilizados para o tratamento.	Revisão narrativa	-	-	Resultados sugerem que um comprimento de onda específico de 658 nm^‡‡^, que se enquadra na faixa da luz vermelha, pode ser mais eficaz no tratamento de LP^||^.
E^¶^4^19^	Avaliar a eficácia da FBM^‡^, do ultrassom e de agentes eletrofísicos de alta frequência na cicatrização de lesões por pressão em adultos e idosos.	Revisão sistemática	(n^†^=12)	-	A FBM^‡^ apresentou eficácia semelhante a outras tecnologias indicadas em outros estudos na cicatrização de lesões por pressão. A FBM^‡^ com comprimento de onda vermelho (660 nm^‡‡^) nos estágios 2 e 3 das lesões por pressão promoveu efetivamente a cura em comparação ao tratamento padrão. Observou-se que o uso da FBM^‡^ acelera o reparo tecidual nas lesões por pressão.
E^¶^5^20^	Investigar a segurança e eficácia do tratamento com PDT^§^ com uso de azul de metileno em lesões que apresentaram sinais de inflamação indicativos de infecção.	Relato de caso	(n^†^=3)	PDT^§^ com azul de metileno na concentração de 10 mg/mL^¶¶^ e fonte de LED^||||^ (660 nm^‡‡^). PDT^§^ aplicada uma vez por semana, por três anos.	A redução de tamanho observada foi extremamente significativa, passando de 26,2 cm*** de comprimento e 9,5 cm*** de largura (248,9 cm^2†††^) para 5,2 cm*** por 4,0 cm*** (20,8 cm^2†††^). Os aspectos de infecção da lesão também diminuíram ao longo do tratamento, com formação de tecido de granulação aderido, porém friável, média drenagem serossanguinolenta e leve odor.
E^¶^6^21^	Relatar as experiências de uma enfermeira especialista na condução de um caso clínico de uma paciente idosa com LP^||^ estágio 3, submetida a terapia de laser de baixa intensidade.	Relato de experiência	(n^†^=1)	LLLT^††^ com comprimento de onda vermelho (660 nm^‡‡^) e infravermelho (808 nm^‡‡^) com 1 J^§§^/cm²^†††^ laser vermelho e infravermelho. LLLT^††^ aplicada a cada 48 horas por dois meses.	Na 5ª aplicação do laser observou-se importante melhora na secreção e no eritema perilesional. No período de dois meses de laser e curativos obteve-se a cicatrização completa da lesão.
E^¶^7^22^	Determinar como os fisioterapeutas lidam com pacientes com paraplegia e lesões por pressão sacrais, bem como a eficácia da terapia a laser no tratamento de lesões por pressão grandes e graves.	Relato de caso	(n^†^=1)	LLLT^††^ com luz de GaAlAs^‡‡‡^, feixe contínuo, sem contato (sem pulsação) no comprimento de onda de 658 nm^‡‡^ de laser infravermelho. LLLT^††^ aplicada cinco vezes por semana, entre 8 a 15 minutos de irradiação, durante um mês.	O tratamento de lesões por pressão com laserterapia no comprimento de onda de 658 nm^‡‡^ pareceu ser bem-sucedido.
E^¶^8^23^	Avaliar a eficácia de um protótipo de fototerapia LED^||||^ em participantes com lesão por pressão.	Ensaio intervencionista randomizado e controlado por placebo	(n^†^=15)	LLLT^††^ com LED^||||^ de comprimento de onda de 630 nm^‡‡^ (vermelho) e 940 nm^‡‡^ (infravermelho), dose de 6 J^§§^/cm^2†††^ (grupo I) e 8 J^§§^/cm^2†††^ (grupo II). LLLT^††^ aplicada três vezes por semana durante oito semanas, totalizando 24 sessões em cada participante.	A área das LP^||^ apresentou redução estatisticamente significativa (p<0,001) ao longo das 24 sessões em todos os grupos de tratamento. Os grupos que receberam fototerapia LED^||||^ (I e II) apresentaram maior cicatrização em comparação ao grupo III (placebo). A taxa de cicatrização foi maior no grupo II, variando de 94,5% a 98,7% na 24ª sessão.
E^¶^9^24^	Examinar a etiologia de lesões que não cicatrizam e diferentes tratamentos para o manejo de lesões.	Revisão narrativa	-	-	Os comprimentos de onda de 633-904 nm^‡‡^ causam a cura mais rápida de LP^||^. A irradiação por meio da LLLT^††^ também induz a cura pelo seu efeito antibacteriano. Embora 650 nm^‡‡^ seja mais eficaz, a carga bacteriana diminui sob o tratamento com comprimento de onda de 830 e 904 nm^‡‡^, mas aumenta sob o comprimento de onda de 670 nm^‡‡^.
E^¶^10^25^	Avaliar a utilidade da SSIT^§§§^ como ferramenta prognóstica no tratamento de LP^||^ estágios III e IV, com curativos de hidrocoloide/hidrogel mais 20 exposições à LLLT^††^, em comparação com curativos hidrocoloides sozinhos, em um grupo de pacientes acamados de longa permanência.	Estudo comparativo aleatorizado	(n^†^=43)	LLLT^††^ com comprimento de onda de 808 nm^‡‡^. Grupo I: LP^||^ tratadas com curativos especializados e terapia com laser (cinco vezes por semana por quatro semanas); Grupo II: LP^||^ tratadas com curativos especializados sem terapia com laser.	No estudo foram observadas três variantes de cicatrização de LP^||^: cicatrização pura com granulação mínima; cicatrização com hipergranulação; e não cicatrizante. As análises dos padrões termográficos relacionados à SSIT^§§§^ revelaram sua dependência do curso da cura. A porcentagem de sucesso na cicatrização de LP^||^ atingiu 79,2% no grupo I em comparação com 73,7% no grupo II (p<0,05). A variante dominante de cicatrização no Grupo I foi a cicatrização pura com granulação mínima, enquanto no grupo II as variantes cicatrização pura com granulação mínima e cicatrização com hipergranulação estiveram presentes com igual frequência.
E^¶^11^26^	Realizar uma revisão sistemática avaliando a eficácia da FBM^‡^ em forma de LLLT^††^ no tratamento de lesões por pressão em adultos e idosos.	Revisão sistemática	(n^†^=5)	-	A FBM^‡^ no comprimento de onda infravermelho apresentou eficácia na cicatrização da lesão por pressão, semelhante ao cuidado padrão apresentado nos diferentes estudos. O FBM^‡^ (658 nm^‡‡^) foi eficaz na promoção da cura quando comparado ao tratamento padrão.
E^¶^12^27^	Fornecer antecedentes e examinar evidências para a aplicação terapêutica de tratamentos de energia luminosa para cicatrização de lesões.	Revisão narrativa	-	-	O tratamento com laser de 658 nm^‡‡^ foi mais eficaz (70% de fechamento, p< 0,05) na promoção do fechamento de LP^||^. Em contraste, os tratamentos com laser de 808 e 940 nm^‡‡^ (fechamento de 31% e 30%, respectivamente) não pareceram melhorar significativamente as taxas de cura em comparação com o grupo placebo (fechamento de 28%).
E^¶^13^28^	Estudar a eficácia da laserterapia no processo de cicatrização de lesões por pressão.	Revisão sistemática	(n^†^=11)	-	Destaca-se que doses de 4 J^§§^/cm^2†††^ com comprimento de onda de 658 nm^‡‡^ foram as mais eficazes no tratamento das lesões por pressão.
E^¶^14^29^	Avaliar o efeito da irradiação laser em diferentes comprimentos de onda na expressão de fatores de crescimento e mediadores inflamatórios selecionados em fases específicas do processo de cicatrização de lesões. Foram incluídos no estudo pacientes com diagnóstico de lesão crônica de etiologia relacionada à LP^||^ (II, III, IV).	Ensaio clínico randomizado	(n^†^=67)	LLLT^††^ com luz de GaAlAs^‡‡‡^. Grupo A - 940 nm^‡‡^; Grupo C - 658 nm^‡‡^; Grupo B - 808 nm^‡‡^; Grupo D - terapia simulada. Tratados com LLLT^††^ e analisados uma vez ao dia, cinco dias por semana por um mês.	Para o grupo C (658 nm^‡‡^), a alteração na concentração de TNF-α^||||||^ foi mais intensa (redução de aproximadamente 75%), enquanto as alterações em outros grupos não foram tão óbvias (redução de aproximadamente 50%). Parece que o efeito bem-sucedido da cicatrização de lesões após irradiação em comprimentos de onda de 658 nm^‡‡^ está associado a um efeito anti-inflamatório, bem como à estimulação de fenômenos como angiogênese, proliferação ou remodelação de tecidos durante o processo de fechamento da lesão.
E^¶^15^30^	Apresentar um relato de caso de LP^||^ na região do calcâneo em paciente diabético tratado com combinação de FBM^‡^, laserterapia e aplicação de membrana de celulose.	Relato de caso	(n^†^=1)	A LLLT^††^ com laser de 660 nm^‡‡^ (vermelho visível) de forma pontual e contínua; e PDT^§^ (placa de 30 LEDs^||||^ de comprimento de onda de 450±10 nm^‡‡^ azul visível por 12 minutos com uma irradiância de 30 mW^¶¶¶^/cm^2†††^) e agente fotossensível curcumina de 1,5%. LLLT^††^ aplicada duas vezes por semana e 1 sessão de PDT^§^.	A cicatrização da LP^||^ ocorreu após 30 dias de tratamento após observação da epitelização total da lesão.
E^¶^16^31^	Comparar a ação dos lasers HeNe**** e AsGa^††††^ no processo cicatricial de LP^||^ e desenvolver um trabalho com medidas de prevenção como tratamento.	Relato de caso	(n^†^=3)	Caso I - LLLT^††^ com laser de HeNe****; Casos II e III - LLLT^††^ com laser de AsGa^††††^. Caso I - foram totalizadas 52 sessões de LLLT^††^ de HeNe****; Casos II e III - 22 a 46 sessões de com LLLT^††^ de AsGa^††††^.	Paciente 1 - ao término das aplicações, observou-se redução da lesão em 100% e relato de melhora consubstancial na qualidade de vida do usuário. Paciente 2 - completa cicatrização da lesão em 22 sessões. Paciente 3 - houve uma redução na profundidade da lesão para 0,5 cm***, mantendo-se os tamanhos de altura e largura. Observou-se pouca melhora na cicatrização da lesão por pressão, porém uma pequena melhora no aspecto da referida lesão.
E^¶^17^32^	Avaliar os efeitos da LLLT^††^ em LP^||^ em humanos por meio de uma revisão sistemática de estudos randomizados.	Revisão sistemática	(n^†^=4)	-	Resultados significativos foram observados no uso de LLLT^††^ com comprimento de onda de 658 nm^‡‡^, e nenhuma evidência foi encontrada para uso de comprimentos de onda acima daquele para o tratamento de LP^||^.
E^¶^18^33^	Descrever o processo de cicatrização de lesão por pressão em paciente crítico tratado com terapêutica convencional de curativo acrescida de laserterapia de baixa intensidade avaliada pela PUSH^‡‡‡‡^ e pelo resultado cicatrização de lesões: segunda intenção, da NOC^§§§§^.	Relato de caso	(n^†^=1)	LLLT^††^ com o laser AlGaInP^||||||||^, comprimento de onda de 660 nm^‡‡^. LLLT^††^ aplicada uma vez ao dia, três vezes na semana, por um período de cinco semanas consecutivas, totalizando 15 aplicações.	Destaca-se a redução da lesão de 7 cm*** de comprimento para 1,5 cm*** e de 6 cm*** de largura para 1,1 cm***, comparando-se o primeiro e o 15º dia de avaliação. O tecido epitelial permanecia em ascendência, com diminuição significativa da quantidade de secreção serossanguinolenta e ausência de odor fétido. Porém o eritema e a maceração perilesional apresentavam leve piora, provavelmente, pela diurese em fraldas, que aumentou a umidade perineal.
E^¶^19^34^	Resumir e avaliar criticamente as evidências das Revisões Sistemáticas sobre intervenções não farmacológicas para tratar lesões de pressão em pacientes idosos.	Revisão narrativa	n^†^=110	-	A LLLT^††^ e a iluminação por LED^||||^ são usadas em vários tipos de lesões incluindo cicatrização de lesões que não cicatrizam, como lesões de pé diabético, lesões de pressão, lesões venosas e pós-quimioterapia contra lesões de radiação. Em estudos comparativos, a exposição de lesões de pressão crónicas que não cicatrizam e que não respondem aos cuidados médicos padrão a comprimentos de onda na gama de 635 e 810 nm^‡‡^ resulta na sua cicatrização mais rápida.
E^¶^20^35^	Examinar pesquisas relacionadas à indução de cicatrização de lesões e por pressão, ativação de genes e eliminação da dor pela aplicação de fotobiomodulação e seus mecanismos de ação.	Revisão narrativa	-	-	Pessoas com LP^||^ grau II em 12 semanas de FBM^‡^ de pulso monocromático apresentam redução no tamanho da lesão de 80 *versus* 50% para pacientes do grupo controle, e a irradiação com 660 e 880 nm^‡‡^ reduz a área inicial da lesão em 13 vezes *versus* indivíduos controles.
E^¶^21^36^	Descrever o tratamento de lesão por pressão crônica em paciente com lesão medular utilizando LLLT^††^	Relato de caso	(n^†^=1)	LLLT^††^ com comprimento de onda de 980 nm^‡‡^ contínuo 6 J^§§^/cm^2†††^ para margens e 655 nm^‡‡^, contínuo 1,8 J^§§^/cm^2†††^ para leito da lesão juntamente com laserterapia intravascular. LLLT^††^ aplicada em dias alternados durante 12 sessões e após aplicada duas vezes por semana, totalizando 24 sessões.	Após duas sessões de laserterapia, a perfusão da lesão melhorou e após a 12ª sessão o diâmetro da lesão reduziu para 3×5 cm^2†††^ com profundidade de 1 cm***. Após 24 sessões de LLLT^††^ e cirurgia de zetaplastia, a lesão cicatrizou completamente.
E^¶^22^37^	Resumir e avaliar criticamente as evidências das revisões sistemáticas dos estudos primários sobre intervenções não farmacológicas para tratar LP^||^ em pacientes idosos.	Revisão sistemática	(n^†^=45)	-	O grau de evidência é muito baixo ou insuficiente para apoiar o uso de terapia adjuvante (ultrassom, pressão negativa, laser, eletromagnética, luz, ondas de choque, hidroterapia, radiofrequência ou terapia vibratória) para aumentar as taxas de cicatrização de LP^||^ em pacientes idosos.
E^¶^23^38^	Identificar a literatura que relata apenas a LLLT^††^, sem agentes fotodinâmicos, como uma tecnologia antimicrobiana/antibiofilme e determinar seus efeitos na cicatrização de lesões.	Revisão narrativa	-	-	Os pacientes com LP^||^ tratados com luz infravermelha (956 nm^‡‡^) e luz vermelha (637 nm^‡‡^) apresentaram uma taxa de cicatrização 49% maior em comparação com os controles. O tempo necessário para o fechamento da lesão em 50% e 90% foi significativamente reduzido. Após 5 semanas de tratamento, a área média da lesão diminuiu para 10%, enquanto os controles levaram 9 semanas para alcançar esse resultado. São poucos os ensaios clínicos que definem um protocolo padrão para erradicação da infecção por LLLT^††^. A partir dos estudos piloto disponíveis, a exposição do paciente à luz de 870 nm^‡‡^/930 nm^‡‡^ e doses de energia superiores a 100 J^§§^/cm^2†††^ parece ser uma abordagem terapêutica eficaz.
E^¶^24^39^	Verificar o feito da LLLT^††^ na forma de LED^||||^ em uma lesão crônica que não cicatriza com duração de 6 meses em um paciente do sexo masculino de 18 anos que sofre de talassemia.	Relato de caso	(n^†^=1)	A LLLT^††^ na forma de LED^||||^. LLLT^††^ com dosagem de 17,3 J^§§^/cm^2†††^ por 8 minutos por 2 semanas seguida de dosagem proliferativa de 8,65-4,33 J^§§^/cm^2†††^ por 4 minutos da 3ª semana até a 6ª semana.	Na 6ª semana de aplicação houve fechamento da lesão.

*Id = Identificação; ^†^n = Número de participantes; ^‡^FBM = Fotobiomodulação; ^§^PDT = Fotodinâmica; ^||^LP = Lesão por pressão; ^¶^E = Estudo; **COVID-19 = *Corona Virus Disease* 2019; ^††^LLLT = *Low-level light therapy*; ^‡‡^nm = Nanômetro; ^§§^J = *Joule*; ^||||^LED = *Light-emitting diode;* ^¶¶^mg/mL = Miligramas por mililitro; ***cm = Centímetros; ^†††^cm^2^ = Centímetros quadrados; ^‡‡‡^GaAlAs = Arseneto de gálio-alumínio; ^§§§^SSIT = *Infrared thermography of the skin surface*; ^||||||^TNF-α = *Tumor necrosis factor alpha*; ^¶¶¶^mW = Miliwatt; ****HeNe = Hélio-neônio; ^††††^AsGa = Arseneto de gálio; ^‡‡‡‡^PUSH *= Pressure Ulcer Scale for Healing*; ^§§§§^NOC *= Nursing Outcomes Classification*; ^||||||||^AlGaInP = Alumínio-gálio-índio-fósforo

## Discussão

A terapia com laser de 808 nm resultou em uma variante dominante de cicatrização pura (H), enquanto o laser de 658 nm mostrou uma redução mais significativa na concentração de TNF-α, sugerindo um efeito anti-inflamatório e estimulante do processo de cicatrização^25,29^. 

Observa-se uma diversidade de tipos de lasers utilizados na LLLT, conforme evidenciado na Figura 4. Em um relato de caso^22^ onde o laser foi aplicado cinco vezes por semana, entre 8 a 15 minutos de irradiação, durante um mês, e em um ensaio clínico randomizado^29^ com 67 pacientes que foram tratados e analisados uma vez ao dia, cinco dias por semana por um mês, empregou-se o laser de Arseneto de Gálio e Alumínio (GaAlAs), um tipo de laser semicondutor que foi introduzido no mercado por volta de 1987^40^. Em outro relato de caso^33^, realizaram-se três aplicações na semana, totalizando 15 aplicações, utilizando o laser Alumínio-Gálio-Índio-Fósforo (AlGaInP). Adicionalmente, no estudo de relato de caso^31^ que acompanhou três pacientes, empregou-se o laser de Hélio-Neônio (HeNe), pertencentes à primeira geração de lasers desenvolvida entre 1975 e 1985, e o laser de Arseneto de Gálio (AsGa), que foi o primeiro laser de diodo disponível no mercado, por volta de 1985^40^. 

Além disso, o ensaio intervencionista randomizado e controlado por placebo^23^, com 15 participantes, que receberam aplicações de laser três vezes por semana durante oito semanas, totalizando 24 sessões, evidenciou uma redução estatisticamente significativa na área das LPs ao longo das sessões de fototerapia com *light-emitting diode* (LED), destacando-se o grupo tratado com LED vermelho, que apresentou a maior taxa de cicatrização. Esses achados sugerem que diferentes comprimentos de onda de laser e LED podem influenciar a cicatrização de LP de maneiras distintas, enfatizando a importância de selecionar o tratamento mais adequado para cada caso clínico. 

O período compreendido entre 1995 e 2005, conhecido como terceira geração, testemunhou a introdução do LED, um dispositivo semicondutor com comprimentos de onda variando de 180 nm a 1 mm. Esses dispositivos, principalmente produzidos pelo processo de emissão espontânea, foram concebidos com a proposta de serem economicamente mais acessíveis e de fácil acionamento eletrônico^40^. Este tipo também foi empregado em três relatos de casos^20,30,39^, que evidenciaram bons resultados, de modo que em um deles a lesão cicatrizou em 6 semanas^39^, outro após 30 dias^30^ e no terceiro apresentou redução significativa do tamanho da lesão e melhora do aspecto da lesão^20^. Nos dois últimos, foi empregada a PDT, com o uso de fotossensibilizadores, como o azul de metileno a 1% de concentração^20^ e a curcumina a 1,5%^30^, resultando em redução do tamanho da lesão e formação de tecido de granulação.

Os estudos de caso revisados apresentam uma série de resultados encorajadores no uso da laserterapia no tratamento de LP. Em primeiro lugar, destaca-se a eficácia de diferentes protocolos de laserterapia na redução das lesões e na melhoria da qualidade de vida dos pacientes^31^. Além disso, a laserterapia mostrou-se eficaz na redução do tamanho das lesões, melhora do tecido epitelial e diminuição dos aspectos de infecção da lesão^20,22,33^.

Outro ponto comum entre os estudos foi a observação da completa cicatrização das LPs após um número variável de sessões de laserterapia, indicando um potencial significativo dessa abordagem terapêutica^21,36,39^. 

Os resultados também indicaram que a laserterapia pode promover cicatrização por segunda intenção, com redução do tamanho da lesão e melhoria dos indicadores clínicos, como presença de tecido de epitelização e ausência de exsudato^16,30^. Esses achados sugerem que a laserterapia pode desempenhar um papel importante na reparação tecidual e no manejo de lesões por pressão.

Além dos achados mencionados, é importante observar que os estudos revisados utilizaram uma variedade de comprimentos de onda na LLLT. Comprimentos de onda comuns incluem 658 nm, 660 nm, 808 nm e 980 nm, cada um com propriedades específicas que podem influenciar os resultados terapêuticos.

As revisões narrativas e sistemáticas destacam a eficácia de diferentes comprimentos de onda na LLLT para o tratamento de LP. O comprimento de onda de 658 nm emerge como um dos mais eficazes, demonstrando uma taxa de fechamento significativamente maior em comparação com outros comprimentos de onda, como 808 nm e 940 nm^27^. Além disso, a luz vermelha, especialmente na faixa de comprimento de onda de 633-904 nm, mostrou resultados promissores na aceleração da cicatrização de LP^17,24^. A terapia com luz infravermelha, especificamente em 637 nm, também apresentou uma taxa de cicatrização significativamente maior em comparação com os grupos controles^38^.

Por outro lado, as revisões sistemáticas destacam a efetividade do laser de 658 nm em doses específicas, como 4 J/cm^2^, para o tratamento de LP^28,32^. Além disso, a FBM com comprimento de onda vermelho (660 nm) nos estágios 2 e 3 das LPs^19^ promoveu efetivamente a cura em comparação ao tratamento padrão^19,26^. No entanto, evidências sobre terapias adjuvantes, como laserterapia, para aumentar as taxas de cicatrização de LP em pacientes idosos são limitadas e de qualidade muito baixa^37^.

Destaca-se ainda que, de acordo com as revisões, os comprimentos de onda de 650 nm, 660 nm e 880 nm apresentaram resultados promissores na redução do tamanho inicial da LP e na promoção da cicatrização^18,34-35^. Esses achados reforçam a importância de considerar uma variedade de comprimentos de onda na terapia com laser para LP, ressaltando a necessidade de mais pesquisas para desenvolver protocolos terapêuticos padronizados e diretrizes de tratamento.

É importante destacar que o presente estudo buscou mapear as evidências disponíveis sobre a FBM e PDT no tratamento de LP, mostrando que as mesmas apresentam resultados promissores na cicatrização, redução do tamanho das lesões e melhoria dos indicadores clínicos no tratamento de LP. Assim, os resultados deste estudo podem contribuir para a formulação de protocolos clínicos, orientando o investimento na inovação tecnológica em saúde e, consequentemente, contribuindo com a qualidade de vida das pessoas com LP. 

É imperativo ressaltar como uma limitação da pesquisa a escassez de estudos clínicos robustos *in vivo* sobre o fenômeno investigado, o que resulta em lacunas significativas em relação aos parâmetros mais confiáveis para a utilização das terapias de FBM e PDT no tratamento de LP. Dessa forma, torna-se imprescindível um maior investimento em pesquisas que possam estabelecer diretrizes claras e protocolos padronizados, a fim de orientar a prática clínica de maneira mais sólida e maximizar os benefícios terapêuticos dessas terapias.

## Conclusão

Em síntese, os estudos revisados proporcionam uma visão abrangente sobre o efeito da FBM e PDT no tratamento de LP, destacando resultados promissores em termos de cicatrização, redução do tamanho das lesões e melhoria dos indicadores clínicos. A diversidade de abordagens terapêuticas disponíveis, incluindo diferentes comprimentos de onda e protocolos de tratamento, ressalta a importância de uma abordagem personalizada para otimizar os resultados clínicos.

É importante destacar que, apesar dos avanços promissores, ainda existem desafios a serem enfrentados na aplicação clínica dessas terapias. A falta de consenso quanto aos parâmetros ideais de tratamento, como a escolha do comprimento de onda mais adequado e a dosagem ideal de energia, representa uma barreira significativa. Além disso, a heterogeneidade dos estudos e a falta de padronização nos métodos de avaliação dos resultados dificultam a comparação entre os diferentes ensaios clínicos. Essas questões ressaltam a necessidade premente de pesquisas adicionais, bem como de colaboração interdisciplinar entre profissionais da saúde, para aprimorar a eficácia e a aplicabilidade clínica da FBM e PDT em LP.
